# Repeated hypoglycemia remodels neural inputs and disrupts mitochondrial function to blunt glucose-inhibited GHRH neuron responsiveness

**DOI:** 10.1172/jci.insight.133488

**Published:** 2020-11-05

**Authors:** Mitchell Bayne, Alexandra Alvarsson, Kavya Devarakonda, Rosemary Li, Maria Jimenez-Gonzalez, Darline Garibay, Kaetlyn Conner, Merina Varghese, Madhavika N. Serasinghe, Jerry E. Chipuk, Patrick R. Hof, Sarah A. Stanley

**Affiliations:** 1 Diabetes, Obesity and Metabolism Institute,; 2 Nash Family Department of Neuroscience and Friedman Brain Institute, and; 3 Tisch Cancer Institute and Department of Oncological Sciences, Icahn School of Medicine at Mount Sinai, New York, New York, USA.

**Keywords:** Metabolism, Neuroscience, Diabetes

## Abstract

Hypoglycemia is a frequent complication of diabetes, limiting therapy and increasing morbidity and mortality. With recurrent hypoglycemia, the counterregulatory response (CRR) to decreased blood glucose is blunted, resulting in hypoglycemia-associated autonomic failure (HAAF). The mechanisms leading to these blunted effects are only poorly understood. Here, we report, with ISH, IHC, and the tissue-clearing capability of iDISCO+, that growth hormone releasing hormone (GHRH) neurons represent a unique population of arcuate nucleus neurons activated by glucose deprivation in vivo. Repeated glucose deprivation reduces GHRH neuron activation and remodels excitatory and inhibitory inputs to GHRH neurons. We show that low glucose sensing is coupled to GHRH neuron depolarization, decreased ATP production, and mitochondrial fusion. Repeated hypoglycemia attenuates these responses during low glucose. By maintaining mitochondrial length with the small molecule mitochondrial division inhibitor-1, we preserved hypoglycemia sensitivity in vitro and in vivo. Our findings present possible mechanisms for the blunting of the CRR, significantly broaden our understanding of the structure of GHRH neurons, and reveal that mitochondrial dynamics play an important role in HAAF. We conclude that interventions targeting mitochondrial fission in GHRH neurons may offer a new pathway to prevent HAAF in patients with diabetes.

## Abstract 2

GHRH neurons in the arcuate nucleus are activated by glucose deprivation; however, repeated hypoglycemia blunts activation, remodels inputs, and disrupts mitochondrial fusion.

## Graphical abstract

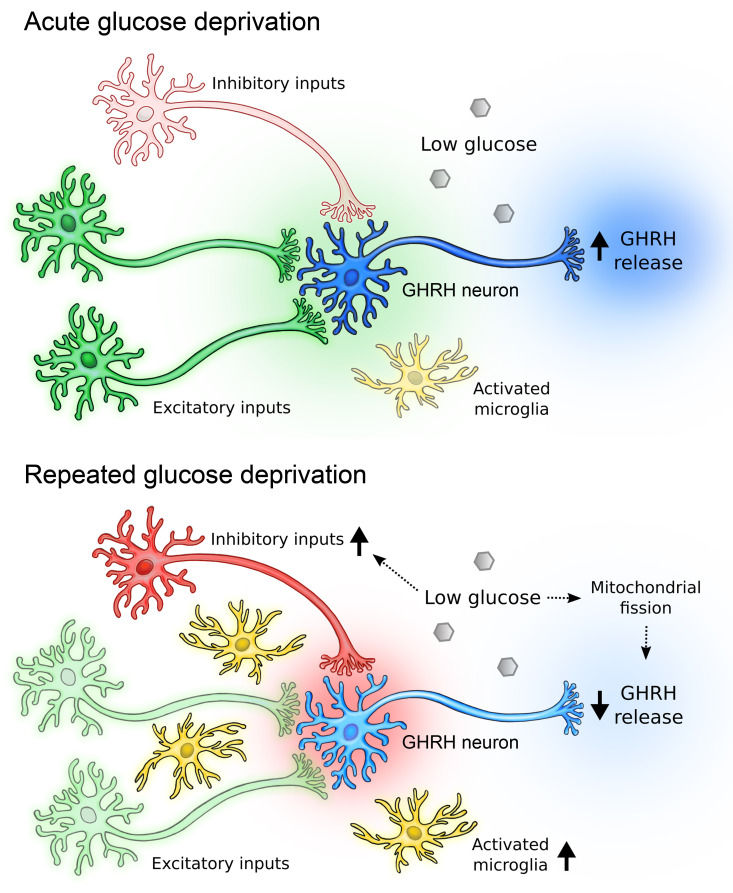

## Introduction

Hypoglycemia is a frequent occurrence for people with diabetes. Intensive blood glucose control (1) ameliorates diabetic complications but poses a major risk of hypoglycemia. Over 80% of individuals with type 1 diabetes and almost half of those with type 2 diabetes experience at least 1 episode of severe hypoglycemia each month (2). In a study using continuous glucose monitoring, individuals with T1D were hypoglycemic (<60 mg/dL) for over 6% of each day (3). Repeated hypoglycemia blunts the release of counterregulatory hormones and diminishes the physiological responses to subsequent hypoglycemia, known as hypoglycemia-associated autonomic failure (HAAF) (4). In turn, HAAF increases the risk of severe hypoglycemia by approximately 20-fold and significantly increases morbidity and mortality (5). The risks of hypoglycemia limit effective therapy (6) and reduce well-being in many individuals with diabetes (7).

Under normal circumstances, plasma glucose levels are maintained within a narrow range. In healthy individuals, hypoglycemia rapidly triggers the counterregulatory response (CRR) to restore blood glucose (8). These responses occur via direct actions of low glucose on peripheral organs such as the pancreas and indirectly via the central nervous system (CNS). CNS effects include altered pancreatic hormone release, sympathetic activation, increased pituitary (growth hormone, GH; adrenocorticotrophin hormone) and adrenal (corticosterone/cortisol) hormone release, and behavioral responses, such as feeding.

Peripheral and central glucose sensors detect and respond to changes in plasma glucose to contribute to the CRR (9). In the CNS, specialized glucose-sensing neurons use glucose as a signal and can be activated by high or low glucose, defined as glucose excited (GE) or glucose inhibited (GI), respectively. Glucose-sensing neurons are found in many brain regions, including hypothalamic arcuate nucleus (ARC) (8). The ARC includes both GE and GI neurons, which express putative glucose sensors such as glucokinase (Gck) (10), glucose transporter type 2 (11), and ATP-sensitive potassium channels (12). Disrupting mitochondrial fission or fusion in ARC GE neurons impairs glucose sensing and leads to systemic metabolic abnormalities (13). ARC neurons are also synaptically connected to metabolically active organs such as the pancreas, liver, and adipose tissue (14), and ARC neurons play central roles in feeding, a critical behavioral response to hypoglycemia.

A number of mechanisms may contribute to HAAF: for example, altered glucose transport into the CNS (15), use of alternative fuels (16, 17), CNS actions of counterregulatory hormones (18), modified neurotransmitter release (19), and shifted glucose responses in glucose-sensing neurons (20). However, most brain regions are heterogeneous, composed of glucose-sensing and non-sensing populations and a combination of GE and GI neurons. Such heterogeneity may mask important adaptations in neural subpopulations.

We have previously shown that ARC growth hormone releasing hormone (GHRH) neurons are one subpopulation that express Gck and are relatively homogeneous, as over 80% of GHRH neurons are inhibited by glucose in ex vivo electrophysiological studies (10). Therefore, in this study, we tested the hypothesis that repeated glucose deprivation results in structural and functional adaptation of GI GHRH neurons, leading to inadequate activation with low glucose, which may contribute to HAAF.

We identified GHRH neurons as a distinct population of ARC GABAergic neurons that are activated by glucose deprivation in vivo and provide polysynaptic inputs to the pancreas. We modeled hypoglycemia insensitivity with repeated glucose deprivation in vivo and demonstrated blunted GHRH neuron activation associated with remodeling of excitatory and inhibitory inputs and microglial activation. In vitro, acute glucose deprivation of GHRH neurons elevated intracellular calcium, depolarized neurons, and regulated mitochondrial structure and function. However, repeated glucose deprivation led to hypoglycemia insensitivity, caused neuronal hyperpolarization, and prevented mitochondrial elongation. Blocking mitochondrial fission with the small molecule mitochondrial division inhibitor-1 (mdivi-1) maintained hypoglycemia sensitivity in vitro and in vivo.

These findings suggest that GI GHRH neurons may contribute to several components of the CRR and support our hypothesis that repeated glucose deprivation results in structural and functional remodeling at both circuit and cellular levels, blunting GHRH neuron responses to hypoglycemia. Maintaining mitochondrial length preserved GHRH neuron activation with repeated glucose deprivation, suggesting that interventions targeting mitochondrial structure and/or function may prevent HAAF.

## Results

### GHRH neurons are multipolar neurons with sparse dendritic processes.

**Figure 1:**
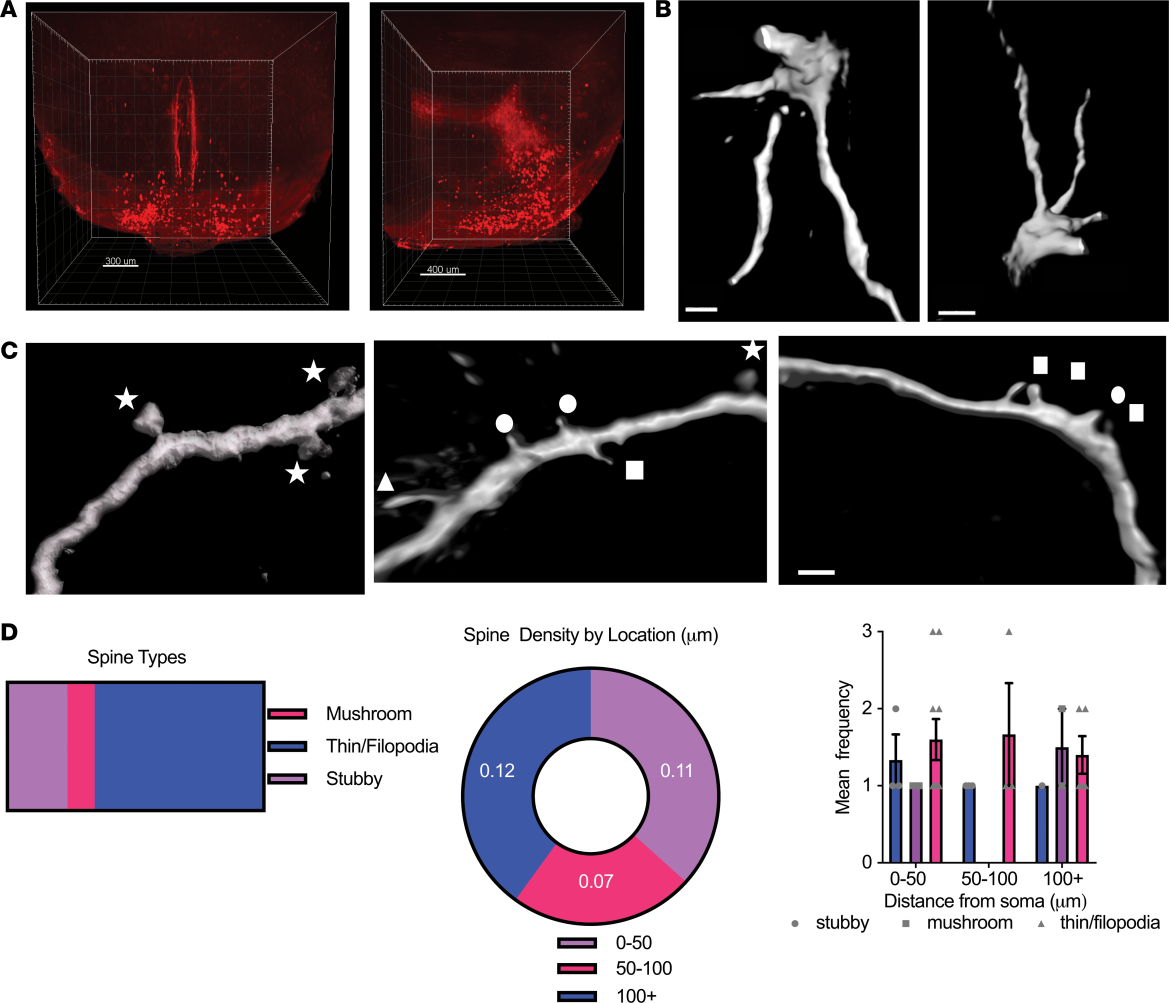
GHRH neurons are multipolar neurons with sparse dendritic processes. (**A**) Light sheet images of brains from GHRH-GFP mice with optical clearing by iDISCO+ showing distribution of GFP^+^ GHRH neurons in coronal and sagittal planes. GFP^+^ neurons are pseudocolored red. (**B**) 3D reconstruction of multipolar GHRH neurons after Alexa Fluor 555 filling and confocal imaging. Scale bars: 2 μm (left), 3 μm (right). (**C**) Maximum-intensity projections of GHRH neurons demonstrating dendritic spine types: thin (rectangles), filopodia (triangle), mushroom (stars), and stubby (circles). Scale bar: 1 μm. (**D**) Proportion of dendritic spine types on filled GHRH neurons (left). Distribution of all dendritic spines with distance from the soma (center) and distribution of specific spine types with distance from the soma (right) (*n* = 47).

We first characterized the distribution and morphology of GHRH neurons using iDISCO+ (21) to clear brains from transgenic mice expressing GFP in GHRH neurons (GHRH-GFP mice) (22) (Figure 1A). GHRH neurons represented a population of 354 ± 10 neurons per brain with expression primarily in the ARC (82.9% ± 4.3%) and a minor population in the periventricular nucleus (14.3% ± 4.3%). Dye-filled ARC GHRH-GFP neurons were uniformly multipolar, with few large dendrites (Figure 1B). GHRH neurons had sparse dendritic spines (0.1 ± 0.02 spines/μm) of which the majority were thin spines or filopodia (65.9%), which can be rapidly remodeled. Stubby (23.4%) and mushroom (10.6%) spines were also present (Figure 1, C and D).

### GHRH neurons are non–agouti related peptide, non–pro-opiomelanocortin GABAergic neurons synaptically connected to the pancreas.

**Figure 2:**
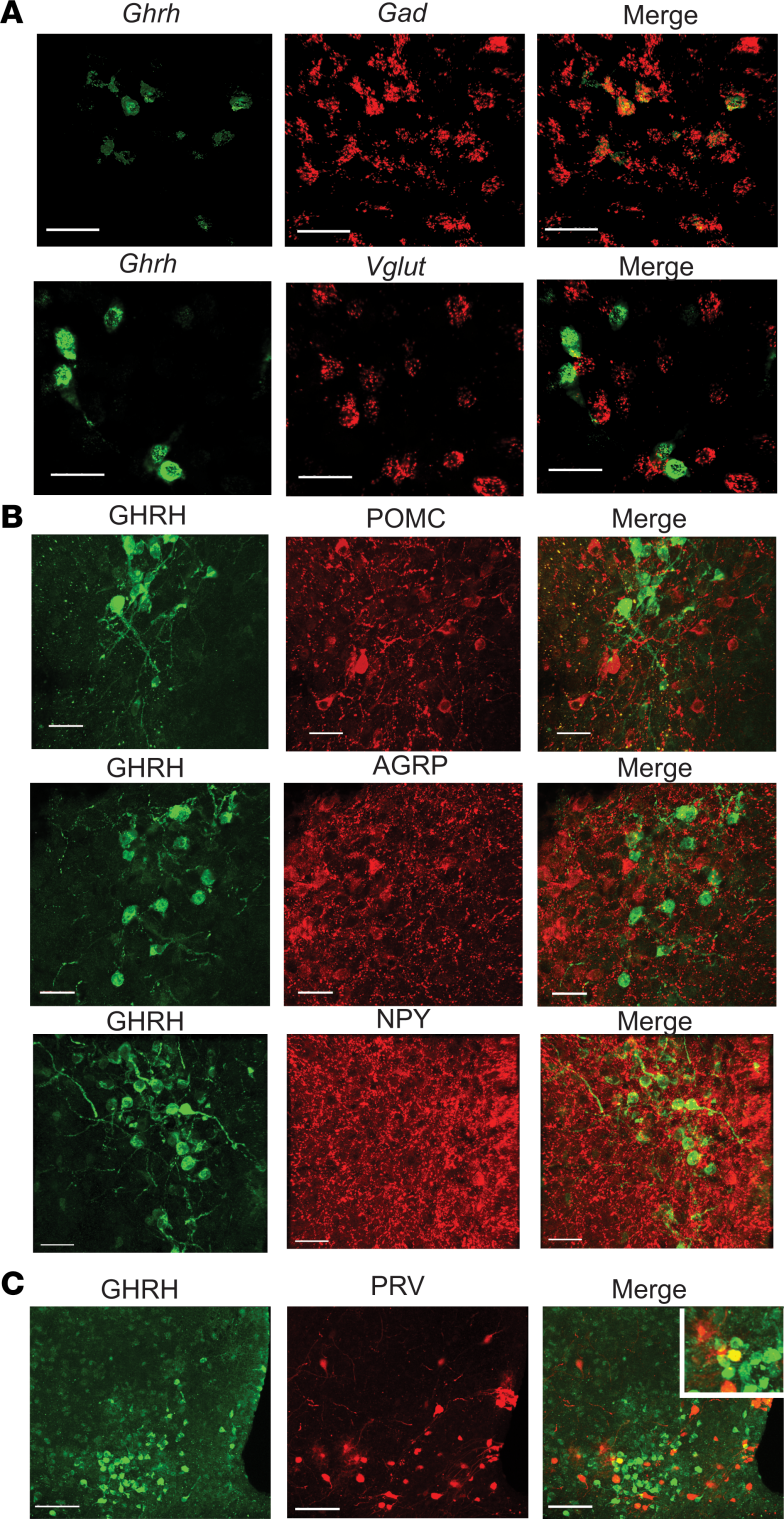
ARC GHRH neurons are non–agouti related peptide, non–pro-opiomelanocortin GABAergic neurons synaptically connected to the pancreas. (**A**) FISH for *GHRH* and *GAD2* (upper) or *VGLUT2* (lower panel) in the ARC (*n* = 4/marker). Scale bar: 30 μm. (**B**) IHC for GFP and pro-opiomelanocortin (POMC, upper), agouti related peptide (AGRP, middle), and neuropeptide Y (NPY, lower panel) in the ARC of GHRH-GFP mice (*n* = 5/marker). Scale bar: 30 μm. (**C**) IHC for GFP and PRV-RFP in the ARC after intrapancreatic injection of PRV-RFP in GHRH-GFP mice. Inset: Overlap between GFP and RFP immunolabeling (*n* = 5). Scale bar: 80 μm**.**

The ARC contains several neural populations that regulate feeding, a crucial response to hypoglycemia. To determine the neurochemical phenotype of GHRH neurons, FISH and IHC labeling approaches were used. FISH for *Vglut2*, *Gad2*, and *Ghrh* revealed GHRH neurons were primarily GABAergic (Figure 2A). Neuropeptide expression was determined by IHC in brain slices from GHRH-GFP mice. Only 0.8% ± 0.5% of GHRH neurons colocalized with neuropeptide Y–expressing (NPY-expressing) neurons, and there was no detectable overlap between GHRH neurons and neurons expressing agouti related peptide (AGRP) or pro-opiomelanocortin (POMC) (Figure 2B).

Next, we investigated if GHRH neurons were synaptically connected to peripheral organs involved in the CRR by injecting the retrograde tracer pseudorabies virus expressing red fluorescent protein (PRV-RFP) into GHRH-GFP mice. A subpopulation of GHRH neurons colocalized with RFP expression after PRV-RFP injection into the pancreas (1.3% ± 0.5% of GHRH-GFP cells, 5.1% ± 1.9% of ARC PRV-RFP–immunoreactive neurons; Figure 2C). There was no overlap between GHRH-GFP–positive neurons and RFP-positive neurons after PRV injection into liver, muscle, epididymal white fat, or adrenal glands.

### Repeated glucose deprivation with 2-deoxy-d-glucose blunts the CRR.

Systemic glucose deprivation was achieved by i.p. administration of 2-deoxy-d-glucose (2DG) (23), a nonmetabolizable glucose analog that mimics the physiological response to hypoglycemia (Supplemental Figure 1A; supplemental material available online with this article; https://doi.org/10.1172/jci.insight.133488DS1). We chose to use 2DG rather than insulin for glucose deprivation because insulin receptors are expressed in ARC neurons (24), and so insulin administration could result in ARC neural activation via receptor binding, independent of glucose deprivation. 2DG injection (400 mg/kg i.p.) resulted in a counterregulatory increase in blood glucose reaching a maximum at 90 minutes; therefore all other measurements were made at this time point. Repeated glucose deprivation (daily 2DG injection for 5 days) blunted the CRR, resulting in a significant decrease in blood glucose after repeated compared with acute 2DG treatment (Supplemental Figure 1B). Acute 2DG treatment significantly increased plasma glucagon, but this effect was reduced by 75% with repeated glucose deprivation (Supplemental Figure 1C). Plasma corticosterone was not altered by repeated glucose deprivation (Supplemental Figure 1D). Despite peripheral hyperglycemia, plasma insulin did not differ between groups, suggesting i.p. 2DG effectively blocked glucose metabolism (25) (Supplemental Figure 1E). Plasma GH was not significantly different between euglycemic mice and those with acute or repeated glucose deprivation. However, GH was significantly higher in mice after repeated glucose deprivation compared with those with acute glucose deprivation (Supplemental Figure 1F).

### Repeated glucose deprivation impairs GHRH neuron activation by acute glucose deprivation.

**Figure 3:**
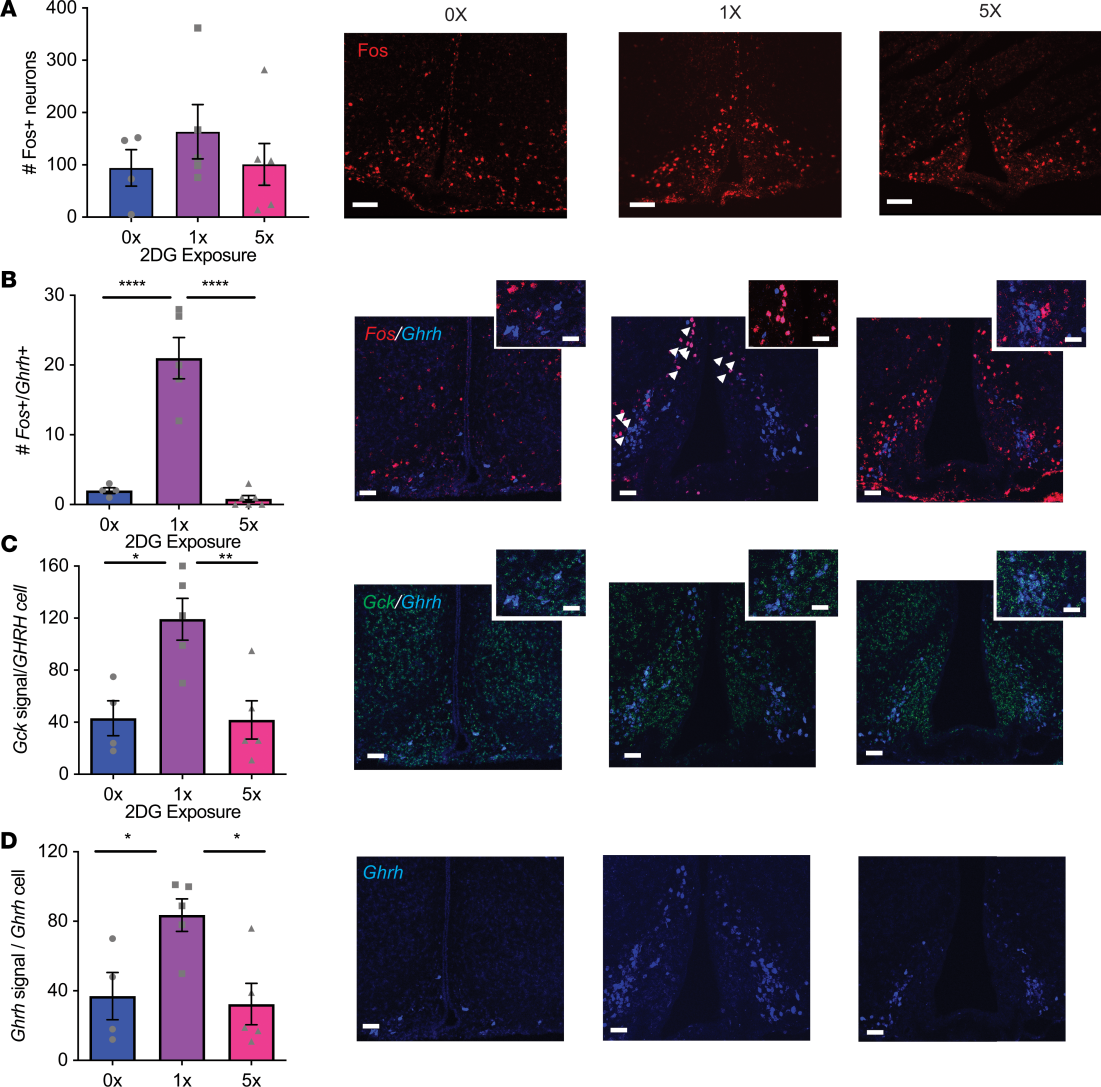
GHRH neuron activation by acute glucose deprivation is impaired by repeated glucose deprivation. Confocal images and quantification of FISH after vehicle (0x) or single (1x) or repeated (5x) i.p. 2DG administration. (**A**) ARC *cfos*; *n* = 4–6/group. (**B**) *Cfos* and *Ghrh*; ****P < 0.0001 vs. 0x and vs. 5x *F*_[2, 12]_ = 42.1, *n* = 4–6/group. Arrowheads indicate neurons’ expression of both *Fos* and *Ghrh*. (**C**) *Gck* and *Ghrh* in the ARC; *P = 0.013 vs. 0x and **P = 0.008 vs. 5x, *F*_[2, 11]_ = 8.94, *n* = 4–5/group. (**D**) *Ghrh* in the ARC; *P = 0.04 vs. 0x and **P = 0.02 vs. 5x, *F*_[2, 11]_ = 6.34, *n* = 4–5/group. One-way ANOVA with Tukey’s multiple-comparisons test. Scale bars: 100 μm (main images), 30 μm (insets). Each dot represents results from individual animals and data are displayed as mean ± SEM.

Previous ex vivo electrophysiological recordings demonstrated GHRH neurons are activated by low glucose (10). We next wanted to validate GHRH neuron activation by glucose deprivation in vivo and test the hypothesis that this activation is blunted by prior glucose deprivation. Acute glucose deprivation increased *Fos*^+^*Ghrh*^+^ neurons by 10-fold, and this effect was abolished by repeated glucose deprivation (Figure 3, A and B). In addition, acute hypoglycemia increased the density of putative glucose sensor *Gck* in GHRH neurons by three-fold and doubled *Ghrh* mRNA probe density compared with vehicle treatment, and this increase was absent with repeated glucose deprivation (Figure 3, C and D). These data are consistent with GHRH neuron activation by glucose deprivation in vivo and suggest rapid upregulation of *Ghrh* and *Gck* expression with low glucose. These effects are lost with repeated glucose deprivation.

### Repeated glucose deprivation alters the morphology of GHRH neurons and activates microglia.

**Figure 4:**
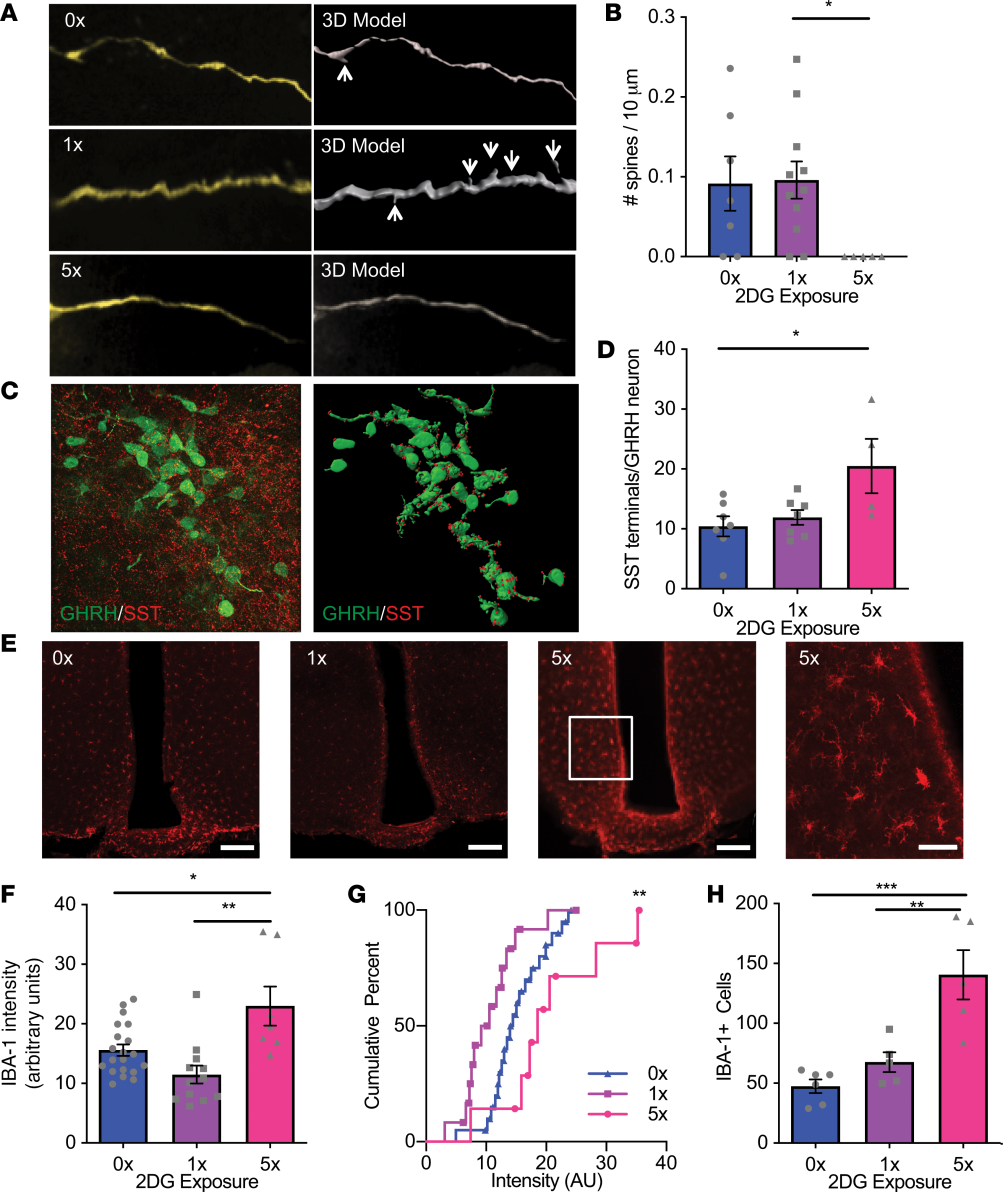
Repeated glucose deprivation disrupts inputs into GHRH neurons and activates microglia. (**A**) Confocal analyses (left) and 3D reconstruction (right) of dendritic spines on Lucifer yellow–filled ARC GHRH-GFP neurons after vehicle (0x) or single (1x) or repeated (5x) i.p. 2DG administration. Scale bar: 5 μm. (**B**) Quantification of dendritic spines on filled ARC GHRH-GFP neurons after vehicle (0x) or single (1x) or repeated (5x) i.p. 2DG administration. *P = 0.03 1x vs. 5x, χ^2^_[2]_ = 7.305, Kruskal-Wallis test with Dunn’s multiple-comparisons test, *n* = 5–11/group. (**C**) Confocal analysis (left) and 3D model (right) of SST terminals contacting ARC GHRH-GFP neurons. Scale bar: 30 μm. (**D**) Quantification of SST terminals contacting ARC GHRH-GFP neurons after vehicle (0x) or single (1x) or repeated (5x) i.p. 2DG administration. *P = 0.02 0x vs. 5x, *F*_[2, 15]_ = 4.87, 1-way ANOVA with Tukey’s multiple-comparisons test, *n* = 4–7/group. (**E**) Confocal analyses of ARC IBA1-positive microglia after vehicle (0x) or single (1x) or repeated (5x) i.p. 2DG administration at low and high magnification. Scale bars: 100 μm for left 3 panels, 50 μm for right panel. (**F**) Quantification of IBA1 intensity by IHC in the ARC after vehicle (0x) or single (1x) or repeated (5x) i.p. 2DG administration. *P = 0.01 0x vs. 5x, **P = 0.0003 1x vs. 5x, *F*_[2, 36]_ = 9.612, 1-way ANOVA with Tukey’s multiple-comparisons test, *n* = 7–20/group. (**G**) Cumulative intensity distribution of IBA1 intensity. **P = 0.028 0x vs. 5x, Kolmogorov-Smirnov test *n* = 7–20/group. (**H**) Quantification of IBA1-positive cells in the ARC after vehicle (0x) or single (1x) or repeated (5x) i.p. 2DG administration. **P = 0.003, ***P = 0.0004, *F*_[2, 13]_ = 15.35, 1-way ANOVA with Tukey’s multiple-comparisons test, *n* = 5–6/group. Each dot represents results from individual animals and data are displayed as mean ± SEM.

Alterations in the balance of excitatory and inhibitory inputs onto GHRH neurons may contribute to low glucose–induced activation of GHRH neurons. Dendritic spines are the primary sites of excitatory glutamatergic synapses in mammalian brains and can undergo remodeling (26). Increased excitatory inputs increase spine number while reduced excitatory inputs and synaptic long-term depression decrease dendritic spine number. Therefore, we quantified dendritic spine number in dye-filled GHRH neurons with acute and repeated glucose deprivation (Figure 4, A and B). Acute glucose deprivation with 2DG did not significantly alter GHRH dendritic spine number compared with vehicle-treated mice. However, dendritic spines on GHRH neurons were undetectable in mice treated with repeated episodes of glucose deprivation. Next, we measured the density of somatostatin-immunoreactive (SST-immunoreactive) terminals, a major inhibitory input, on GHRH neurons. Acute glucose deprivation did not significantly alter SST terminals contacting GHRH neurons, but repeated glucose deprivation doubled SST terminals in apparent apposition with GHRH neurons (Figure 4, C and D). Acute and repeated glucose deprivation did not affect *Fos* expression in ARC *Sst*-expressing neurons. Together, these data suggest GHRH neurons are directly activated by low glucose, but loss of excitatory inputs and increased inhibitory inputs contribute to reduced GHRH neuronal activation with repeated glucose deprivation.

Microglia have been implicated in synaptic pruning (27) and may contribute to remodeling of excitatory and inhibitory inputs into GHRH neurons. To test this hypothesis, we investigated whether acute or repeated glucose deprivation regulates ARC microglia activation, using ionized calcium binding adaptor molecule 1 (IBA1) as a marker of activated microglia (Figure 4E). Acute glucose deprivation did not significantly alter IBA1 expression, but repeated glucose deprivation significantly increased IBA1 immunolabeling intensity (Figure 4, F and G) and the number of IBA1-expressing cells (Figure 4H) compared with acute glucose deprivation. These data are consistent with an increase in microglial activation with repeated glucose deprivation.

**Figure 5:**
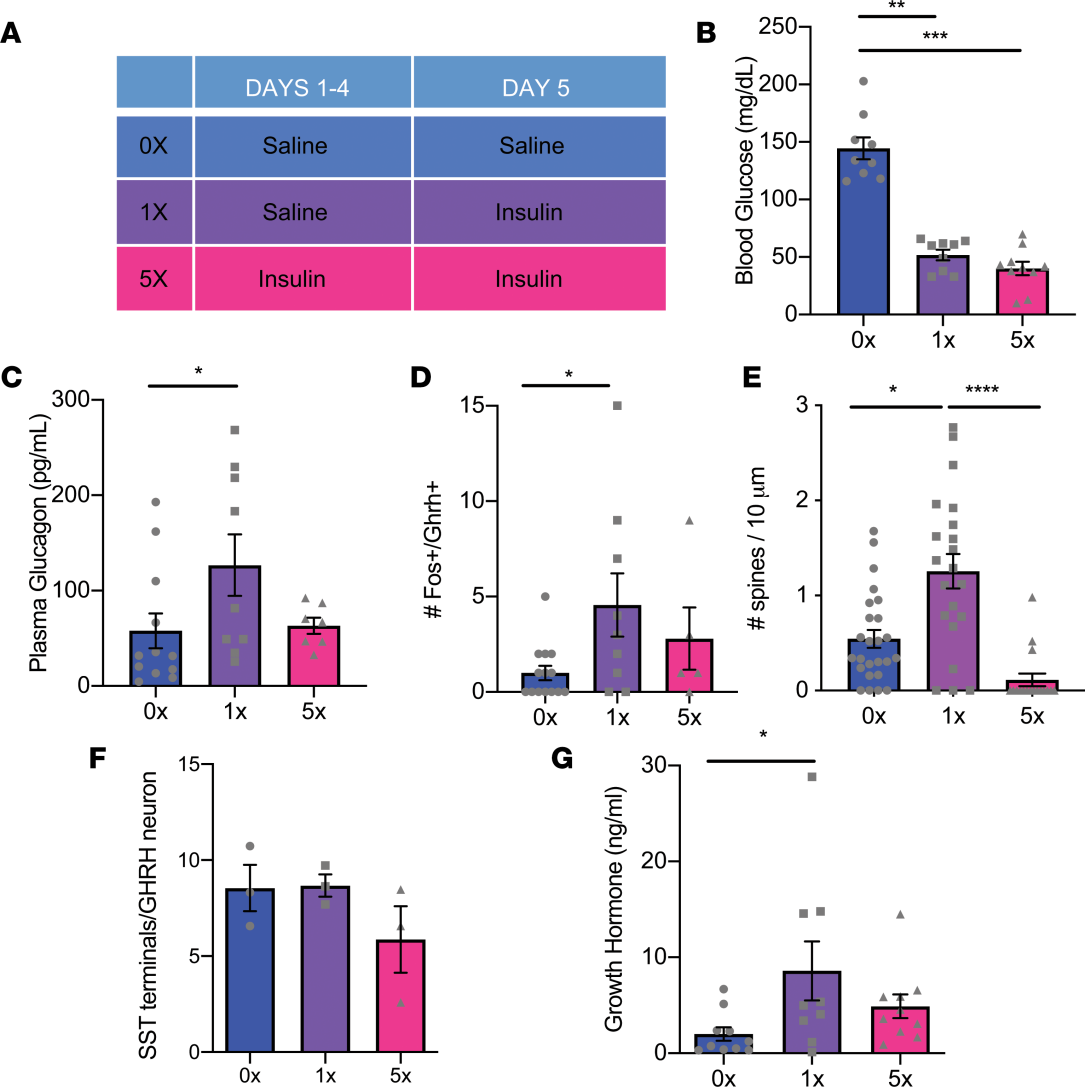
Repeated glucose deprivation with insulin blunts GHRH neuron activation. (**A**) Schema of experimental protocol for repeated glucose deprivation with i.p. administration of insulin in vivo. (**B**) Blood glucose levels (**P < 0.0036, ***P < 0.0002, χ^2^_[2]_ = 18.23, Kruskal-Wallis ANOVA with Dunn’s multiple-comparisons test, *n* = 9–10/group). (**C**) Plasma glucagon levels (*P = 0.04, χ^2^_[2]_ = 5.3, Kruskal-Wallis ANOVA with Dunn’s multiple-comparisons test, *n* = 7–12/group). (**D**) Quantification of fos-positive and GHRH-positive cells in the ARC after vehicle (0x) or single (1x) or repeated (5x) i.p. insulin administration, *n* = 5–14/group. (**E**) Quantification of dendritic spines on ARC GHRH-GFP neurons after vehicle (0x) or single (1x) or repeated (5x) i.p. insulin administration. *P = 0.04 1x vs. 0x; ****P < 0.0001 1x vs. 5x, χ^2^_[2]_ = 25.26 Kruskal-Wallis test with Dunn’s multiple-comparisons test, *n* = 17–21/group. (**F**) Quantification of SST-immunoreactive terminals contacting ARC GHRH-GFP neurons after vehicle (0x) or single (1x) or repeated (5x) i.p. insulin administration, *n* = 3/group. (**G**) Analysis of plasma growth hormone after vehicle (0x) or single (1x) or repeated (5x) i.p. insulin administration. *P = 0.049, *F*_[2, 26]_ = 2.41, 1-way ANOVA with Tukey’s multiple-comparisons test, *n* = 9–10/group.

Because there are differences in the physiological responses to repeated glucose deprivation with 2DG versus repeated insulin-induced hypoglycemia, we assessed GHRH neuron activation, dendritic spine number, inhibitory inputs, and plasma GH in response to repeated insulin-induced hypoglycemia (Figure 5A). Both acute insulin-induced hypoglycemia and glucose deprivation by 2DG activated GHRH neurons, and this effect was absent with repeated 2DG-induced and repeated insulin-induced hypoglycemia (Figure 5D). Similarly, both repeated 2DG and repeated insulin-induced hypoglycemia significantly reduced GHRH dendritic spine density (Figure 5E). These data suggest that these effects are secondary to decreased glucose metabolism. However, there was no significant change in SST-containing terminals contacting GHRH neurons with repeated insulin-induced hypoglycemia (Figure 5F). In contrast to 2DG-induced glucose deprivation and in keeping with clinical testing using insulin-induced hypoglycemia, plasma GH was significantly increased by acute insulin-induced hypoglycemia (mean blood glucose: 51.7 mg/dL; 2.87 mmol/L) (Figure 5, B and G). Although repeated insulin-induced hypoglycemia reduced blood glucose further (mean blood glucose: 40.2 mg/dL; 2.2 mmol/L), there was no significant increase in plasma GH (Figure 5, B and G). Repeated insulin-induced hypoglycemia blunted the corticosterone response (Figure 5C). Differences in the SST inputs into GHRH neurons and plasma GH responses between 2DG and insulin suggest these responses may be related to effects of insulin or peripheral hyperglycemia rather than glucose deprivation.

### Repeated exposure of clonal GHRH neurons to low glucose blunts GHRH release.

**Figure 6:**
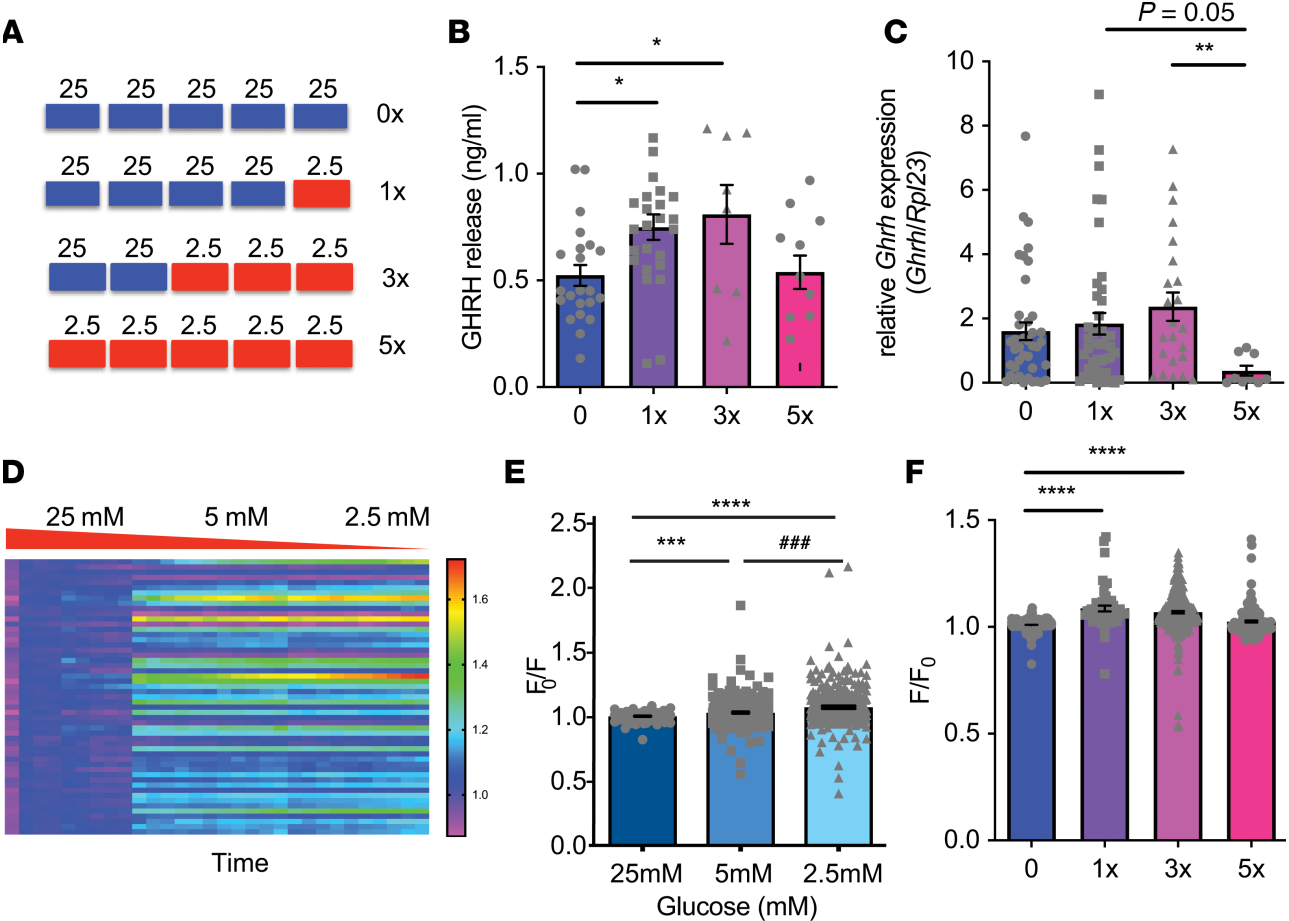
N38 cells are glucose inhibited and responses are blunted by recurrent glucose deprivation. (**A**) Schema of experimental protocol for repeated glucose deprivation of N38 cells in vitro by treatment with media containing 25 mM glucose (standard culture conditions) or 2.5 mM glucose (glucose deprivation). (**B**) Quantification of GHRH release from N38 cells after no (0x) or single (1x) or repeated glucose deprivation (3x and 5x; *n* = 3–4 experiments, in triplicate). *P = 0.04, *F*_[3, 61]_ = 3.854, 1-way ANOVA with Tukey’s multiple-comparisons test. (**C**) Quantification of *Ghrh* expression in N38 cells after no (0x) or single (1x) or repeated glucose deprivation (3x and 5x) (*n* = 3–4 experiments, in triplicate). **P = 0.004, χ^2^_[3]_ = 11.61, Kruskal-Wallis test with Dunn’s multiple-comparisons test. (**D**) Time-resolved calcium responses using calcium indicator Fluo-4 (F/F_0_, color scale) of 53 N38 cells without previous glucose deprivation (1 cell/row) with 25 mM, 5 mM, and 2.5 mM glucose treatment. (**E**) Quantification of peak fluorescence (F/F_0_) with 25 mM, 5 mM, and 2.5 mM glucose treatment in N38 cells without previous glucose deprivation (4 studies, 51–307 cells). ***P = 0.0004 25 mM vs. 5 mM; ^###^*P* = 0.0008 5 mM vs. 2.5 mM; ****P < 0.0001, 25 mM vs. 2.5 mM, χ^2^_[2]_ = 56.2, Kruskal-Wallis test with Dunn’s multiple-comparisons test. (**F**) Quantification of peak fluorescence (F/F_0_) with no (0x) or single (1x) or repeated glucose deprivation (3x and 5x) in N38 cells (4 studies, 170–307 cells). ****P < 0.0001, χ^2^_[3]_ = 188.2, Kruskal-Wallis test with Dunn’s multiple-comparisons test. Each dot represents data from individual cells and data are displayed as mean ± SEM.

Given the complexity of the GHRH-SST-GH system in vivo, we sought to recapitulate blunted hypoglycemia sensing with repeated glucose deprivation using an in vitro system, independent of alterations in excitatory/inhibitory inputs or feedback from circulating hormones (Figure 6A). To do so, we used the mouse embryonic hypothalamic cell line N38. These neurons express *Ghrh* and genes critical to glucose sensing, such as *Gck* and glucose transporters (28). Published studies report that medial basal ARC neurons, such as GHRH neurons, lie outside the blood-brain barrier (29–32) and so are probably exposed to glucose levels equivalent to those in the circulation. We therefore used glucose levels of 2.5 mM (equivalent to those in our in vivo studies) to mimic hypoglycemia. GHRH release from N38 cells was increased by 40%–50% with hypoglycemia (Figure 6B) (1 time and 3 times; 1x and 3x), but this increase was blunted by repeated hypoglycemia (5 times; 5x). *GHRH* mRNA was not significantly increased by a single episode of hypoglycemia, but repeated hypoglycemia (5x) significantly reduced GHRH expression (Figure 6C). These data suggest repeated exposure of N38 cells to low glucose blunts the initial low glucose–induced increase in GHRH release and provides an in vitro model for impaired glucose sensing.

### Repeated exposure of clonal GHRH neurons to low glucose blunts calcium entry and depolarization.

**Figure 7:**
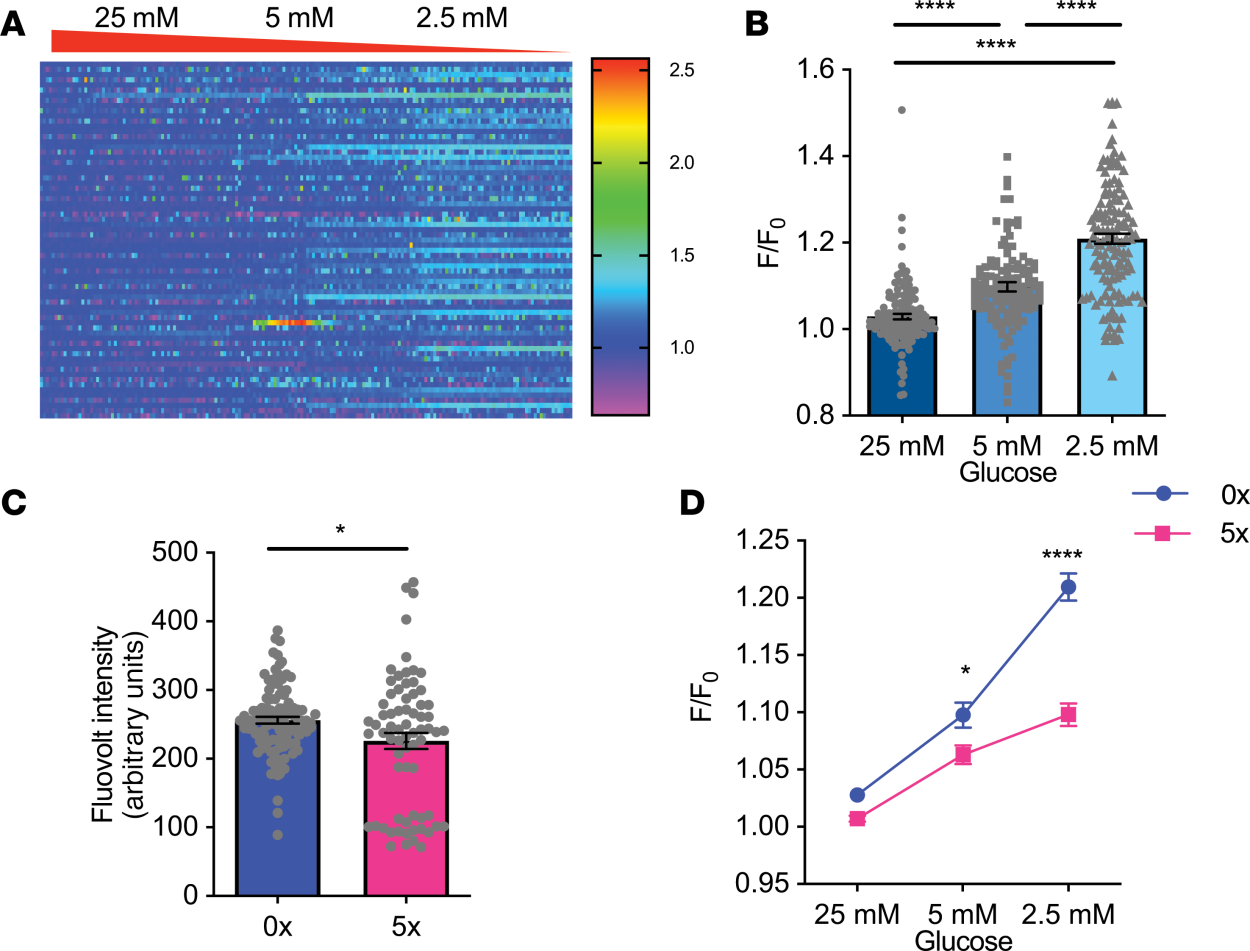
Recurrent glucose deprivation leads to hyperpolarization and impaired depolarization with low glucose. (**A**) Time-resolved voltage responses using voltage indicator Fluovolt (F/F_0_, color scale) of 69 N38 cells without previous glucose deprivation (1 cell per row) with 25 mM, 5 mM, and 2.5 mM glucose treatment. (**B**) Quantification of peak fluorescence (F/F_0_) with 25 mM, 5 mM, and 2.5 mM glucose treatment in N38 cells without previous glucose deprivation (4 studies, 135–144 cells/group). ****P < 0.0001, χ^2^_[2]_ = 153.9, Kruskal-Wallis test with Dunn’s multiple-comparisons test. (**C**) Quantification of basal fluorescence at 25 mM glucose in N38 cells without (0x) and with (5x) previous glucose deprivation (4 studies, 80–105 cells). *P = 0.02, *t* *=* *2*.371, degrees of freedom = 93.5, unpaired *t* test with Welch’s correction. (**D**) Quantification of peak fluorescence (F/F_0_) with 25 mM, 5 mM, and 2.5 mM glucose treatment in N38 cells without previous glucose deprivation (4 studies, 195 cells). *P = 0.03, ****P < 0.0001, significant effect of previous glucose deprivation, *F*_[193, 386]_ = 1.915, *P* < 0.0001, 2-way ANOVA with Holm-Šidák multiple-comparisons test. Each dot represents data from individual cells and data are displayed as mean ± SEM.

Because GHRH release is calcium dependent, we examined the effects of acute or repeated low glucose on intracellular calcium in N38 cells. The intensity of the fluorescent calcium indicator Fluo-4 significantly increased as glucose levels decreased (Figure 6, D and E), indicative of increased intracellular calcium. However, antecedent hypoglycemia significantly blunted the calcium response to low glucose such that it was no longer significantly different from baseline (Figure 6F). Next, we assessed the voltage response to low glucose in N38 cells because voltage-gated calcium channels typically mediate neuronal calcium changes. In keeping with our previous electrophysiological findings in GHRH neurons, N38 cells were depolarized by lowering glucose, as indicated by significantly increased fluorescence intensity of Fluovolt (Figure 7, A and B). However, repeated hypoglycemia (5x) reduced baseline fluorescence intensity by 10%, in keeping with hyperpolarization of N38 cells (Figure 7C), and blunted the depolarization response to low glucose (Figure 7D). Together, these data suggest repeated glucose deprivation hyperpolarizes N38 cells and blunts depolarization in response to low glucose.

### Repeated glucose deprivation blunts low glucose effects on ATP production and mitochondrial length.

**Figure 8:**
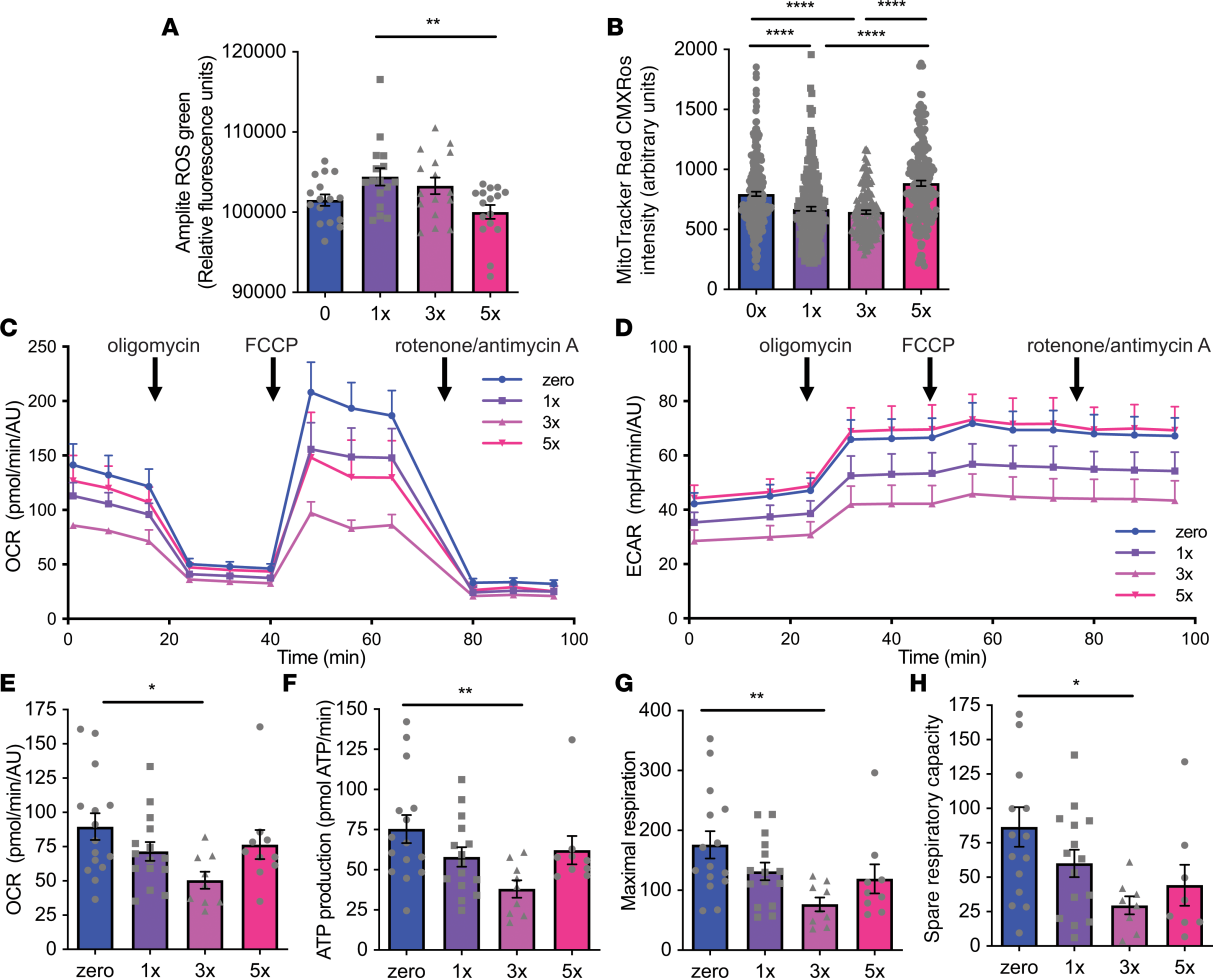
Repeated glucose deprivation blunts the effects of low glucose on mitochondrial function. Quantification in N38 cells after no (0x) or single (1x) or repeated glucose deprivation (3x and 5x). (**A**) Amplite ROS green whole-cell ROS indicator (*n* = 3–4 experiments, in triplicate). **P = 0.008 1x vs. 5x, *F*_[3,__60]_ = 4.246, 1-way ANOVA with Tukey’s multiple-comparisons test. (**B**) MitoTracker Red CMXRos intensity (*n* = 3–4 experiments, in triplicate, 160–252 cells/group). ****P < 0.0001, χ^2^_[3]_ = 83.5, Kruskal-Wallis test with Dunn’s multiple-comparisons test. (**C**) Oxygen consumption rate (OCR) (*n* = 3–4 experiments, in triplicate). FCCP, carbonyl cyanide 4-(trifluoromethoxy)-phenylhydrazone. (**D**) Extracellular acidification rate (ECAR) (*n* = 3–4 experiments, in triplicate). (**E**) Basal respiration rate (*n* = 3–4 experiments, in triplicate). *P = 0.02, χ^2^_[3]_ = 8.665, Kruskal-Wallis test with Dunn’s multiple-comparisons test. (**F**) ATP production (*n* = 3–4 experiments, in triplicate). **P = 0.007, χ^2^_[3]_ = 10.66 Kruskal-Wallis test with Dunn’s multiple-comparisons test. (**G**) Maximal respiration (*n* = 3–4 experiments, in triplicate). **P = 0.001, χ^2^_[3]_ = 12.45, Kruskal-Wallis test with Dunn’s multiple-comparisons test. (**H**) Spare respiratory capacity (*n* = 3–4 experiments, in triplicate). *P = 0.04, χ^2^_[3]_ = 9.015, Kruskal-Wallis test with Dunn’s multiple-comparisons test. Each dot represents data from individual well and data are displayed as mean ± SEM.

Increased reactive oxygen species (ROS) have been proposed to play critical roles in glucose sensing in ventromedial hypothalamus neurons and in ARC GE neurons. Therefore, we measured the effects of acute and repeated low glucose on whole-cell ROS in N38 cells. Repeated glucose deprivation significantly decreased whole-cell ROS, measured by intensity of the fluorescent probe Amplite Fluorescent Green (33), compared with acute deprivation (Figure 8A).

ATP has also been proposed to be a critical intracellular signal for glucose sensing and to play a role in synaptic morphology, which are both disrupted by repeated glucose deprivation. Mitochondrial function is crucial for both ROS and ATP production, so we first assessed mitochondrial membrane potential in response to acute and repeated glucose deprivation. Mitochondrial membrane potential, determined by fluorescence intensity of MitoTracker Red CMXRos, was significantly reduced by acute glucose deprivation but restored by repeated episodes of glucose deprivation (Figure 8B).

Next, we performed bioenergetic profiling in N38 cells after single or repeated episodes of glucose deprivation. Oxygen consumption rate (OCR) and extracellular acidification rate (ECAR) (Figure 8, C–E) progressively declined from control levels with 1 or 3 episodes of glucose deprivation but were partially restored in cells treated with 5 previous episodes of glucose deprivation. Hypoglycemia did not affect nonmitochondrial oxygen consumption (Figure 8C). Acute hypoglycemia (1x or 3x) progressively decreased ATP production and was partially reversed by repeated hypoglycemia (5x) (Figure 8F). Similarly, both maximal respiration and spare respiratory capacity, calculated as the difference between maximal and basal OCR, were progressively decreased by 1 and 3 episodes and partially rescued with 5 episodes of glucose deprivation (Figure 8, G and H). Taken together, these data suggest that repeated glucose deprivation may lead to partial reversal of low glucose–induced changes in ROS and ATP and so impair hypothalamic glucose sensing.

**Figure 9:**
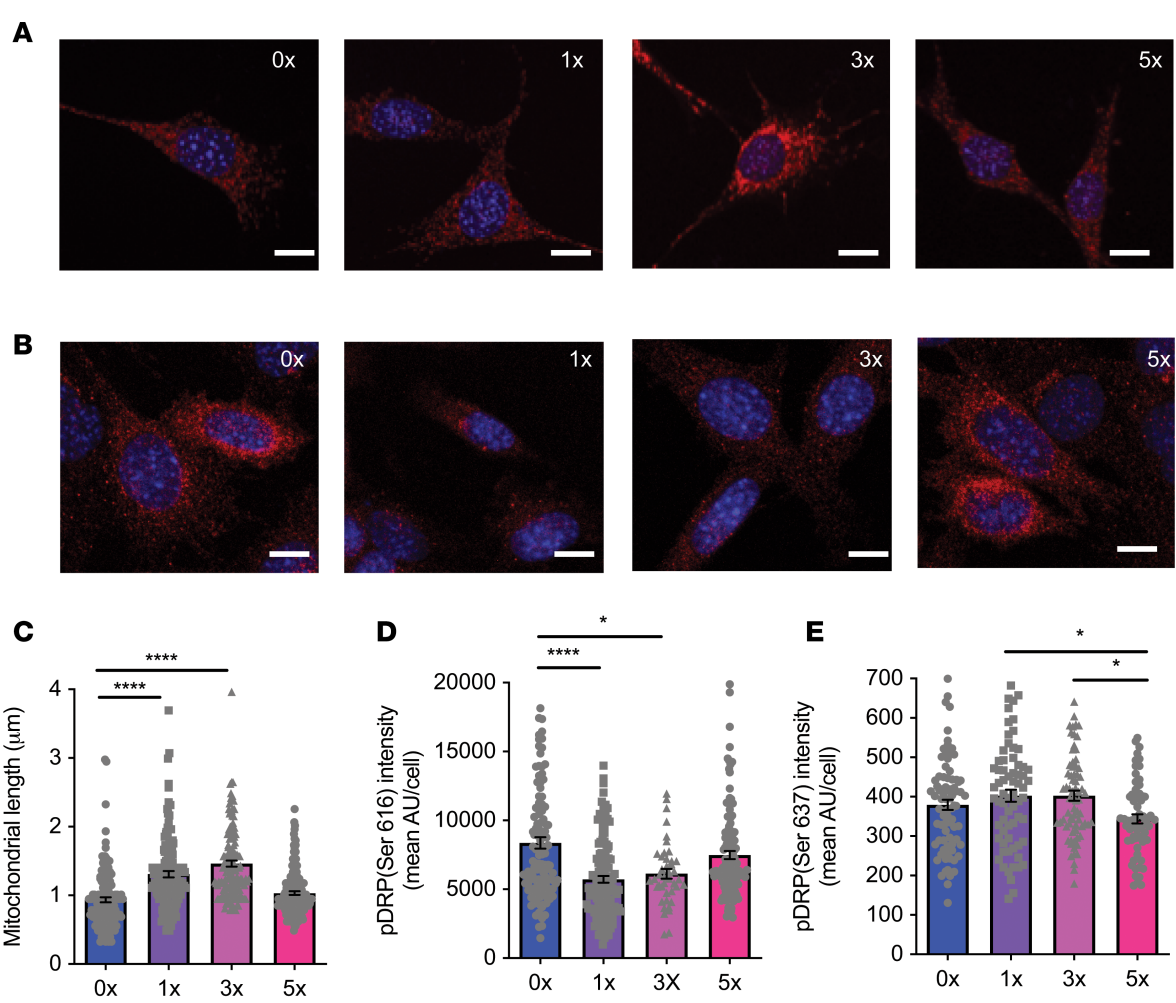
Repeated glucose deprivation blunts effects of low glucose on mitochondrial structure. Confocal images and quantification after no (0x) or single (1x) or repeated glucose deprivation (3x and 5x). (**A**) HSP60 immunostaining of mitochondria in N38 cells. Scale bar: 10 μm. (**B**) p-DRP (Ser616) immunostaining of mitochondria in N38 cells. Scale bar: 10 μm. (**C**) Mitochondrial length in N38 cells (*n* = 4 experiments, 140–160 mitochondria/group). ****P < 0.0001, χ^2^_[3]_ = 125.4, Kruskal-Wallis test with Dunn’s multiple-comparisons test. (**D**) Intensity of p-DRP (Ser616) in N38 cells (*n* = 4 experiments, 40–130 cells/group). *P = 0.03, ****P < 0.0001, χ^2^_[3]_ = 58.7, Kruskal-Wallis test with Dunn’s multiple-comparisons test. (**E**) Intensity of p-DRP (Ser637) in N38 cells (*n* = 4 experiments, 63–81 cells/group). *P = 0.01 vs. 1x and vs. 3x, *F*_[3,__280]_ = 3.875, 1-way ANOVA with Tukey’s multiple-comparisons test. Data are displayed as mean ± SEM.

Mitochondrial function and structure are closely related, so we next examined the effects of glucose deprivation on mitochondrial length, identified using the mitochondrial protein heat shock protein 60 (HSP60) (Figure 9A). Acute glucose deprivation (1x or 3x) increased mitochondrial length by 50%, but this effect was absent after repeated glucose deprivation (5x) (Figure 9, A–C). Phosphorylation of dynamin related protein 1 (p-DRP1), a critical regulator of mitochondrial fission, at Ser616 promotes mitochondrial fission whereas phosphorylation at Ser637 inhibits mitochondrial fission. Acute glucose deprivation (1x or 3x) significantly decreased the intensity of p-DRP1 (Ser616) immunostaining, but further glucose deprivation (5x) blunted this response (Figure 9D). Acute glucose deprivation did not significantly alter p-DRP1 (Ser637) intensity, but repeated glucose deprivation (5x) significantly reduced p-DRP1 (Ser637) intensity compared with acute glucose deprivation (Figure 9E). Therefore, acute low glucose shifts the balance of mitochondrial fission and fusion, leading to elongated mitochondria, which are absent after multiple episodes of low glucose.

The effects of low glucose on N38 cells occur after 5 episodes of low glucose whereas a single episode of hypoglycemia can impair the CRR (34). We therefore tested the effects of 0, 1, and 3 episodes of 2DG treatment in vivo on GHRH neuron activation, dendritic spine density, and SST terminals on GHRH neurons. In keeping with our in vitro results in N38 cells, although blood glucose was blunted by 3 episodes of 2DG treatment, there was no significant change in FOS^+^GHRH^+^ neurons, dendritic spine density, or SST terminals on GHRH neurons with 3 episodes of 2DG (Supplemental Figure 2, A–D). These results suggest that the responses of N38 cells to repeated low glucose mimic those of GHRH neurons to repeated 2DG in vivo and that this circuit may be more resistant to repeated hypoglycemia than other CRRs.

A minor GHRH population lies outside the medial basal ARC and so is protected by the blood-brain barrier and likely to be exposed to lower glucose concentrations than blood glucose. We therefore assessed the effects of repeated hypoglycemia with glucose concentrations similar to those reported in hypothalamic tissue (35, 36) (Supplemental Figure 3A). In line with the effects of 2.5 mM glucose, 0.5 mM glucose increased intracellular calcium, and repeated 0.5 mM glucose deprivation (5x, 0.5 mM) significantly blunted the calcium response to low glucose in N38 cells (Supplemental Figure 3, B–D). Repeated 0.5 mM glucose deprivation had similar effects to repeated 2.5 mM glucose deprivation on mitochondrial length. Acute glucose deprivation (1x or 3x, 0.5 mM) significantly increased mitochondrial length, but this effect was blunted by 5 episodes of glucose deprivation (Supplemental Figure 3E). Acute glucose deprivation (1x, 0.5 mM) significantly decreased the intensity of p-DRP1 (Ser616) immunostaining, but further treatment with low glucose (5x, 0.5 mM) blunted this effect (Supplemental Figure 3F). Repeated glucose deprivation (5x, 0.5 mM) significantly reduced p-DRP1 (Ser637) intensity (Supplemental Figure 3G).

### Mdivi-1 preserves the response to low glucose after repeated glucose deprivation.

**Figure 10:**
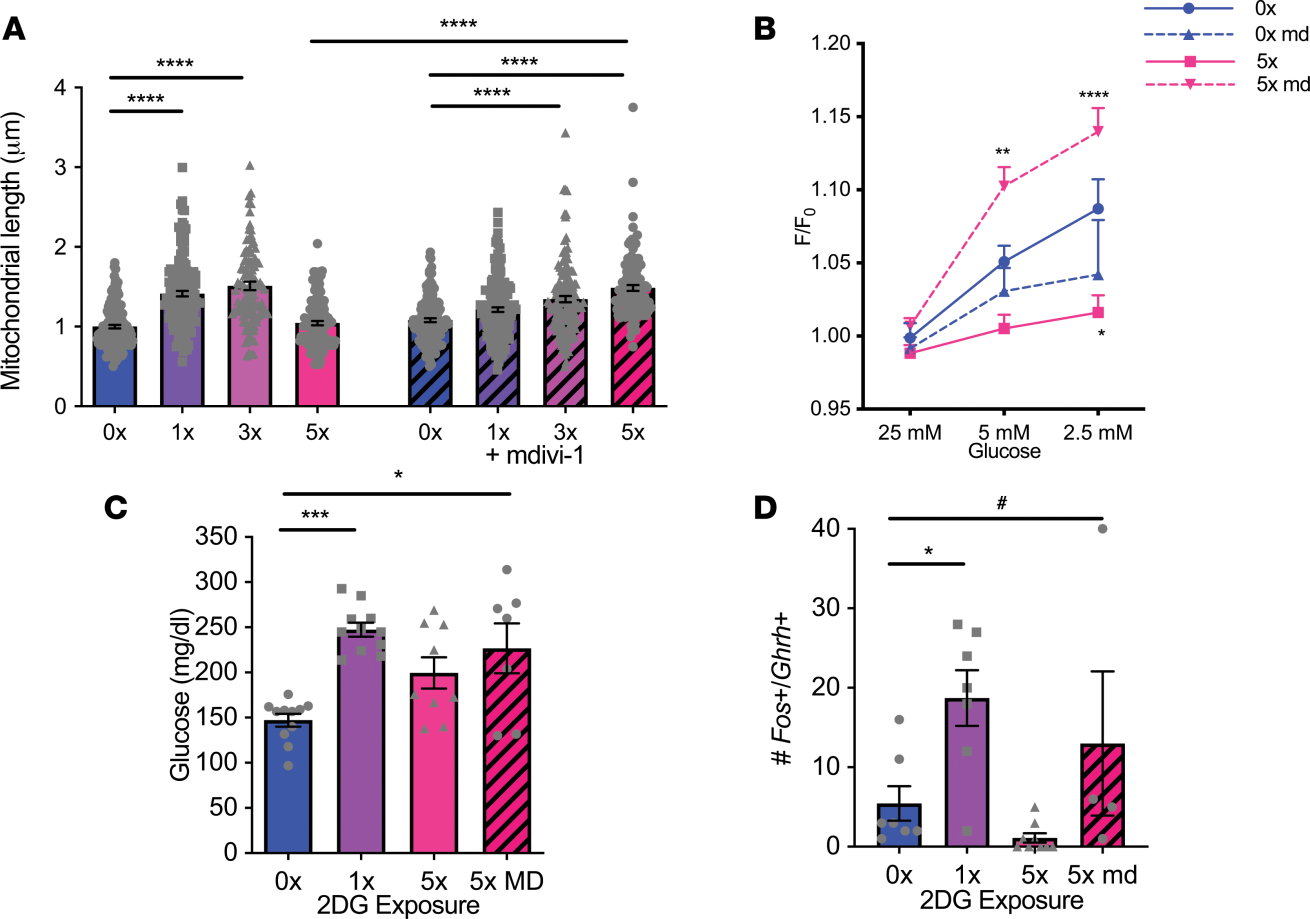
Mdivi-1 preserves the response to low glucose after repeated glucose deprivation. (**A**) Mitochondrial length in N38 cells after no (0x) or single (1x) or repeated glucose deprivation (3x and 5x; *n* = 3 experiments, 110–168 mitochondria/group) with and without mdivi-1 treatment. ****P < 0.0001, *F*_[7, 1082]_ = 35.33, 1-way ANOVA with Tukey’s multiple-comparisons test. (**B**) Peak fluorescence (F/F_0_) with 25 mM, 5 mM, and 2.5 mM glucose treatment in N38 cells with and without previous glucose deprivation, with and without mdivi-1 treatment. ****P < 0.0001 5x with mdivi-1 vs. 5x at 2.5 mM glucose; **P = 0.003 5x with mdivi-1 vs. 5x at 5 mM glucose; *P = 0.01 5x vs. 0x at 2.5 mM glucose. Significant effect of mdivi-1 treatment *P* < 0.0001, *F*_[3, 1482]_ = 9.187, 2-way ANOVA with Tukey’s multiple-comparisons test, *n* = 68–147 cells/group. (**C**) Blood glucose after vehicle (0x) or single (1x) or repeated (5x) i.p. 2DG administration or repeated 2DG administration with mdivi-1 treatment (5x MD). ***P = 0.0004, *P = 0.02, χ^2^_[3]_ = 15.67, Kruskal-Wallis test with Dunn’s multiple-comparisons test, *n* = 7–11/group. (**D**) *c-fos*^+^*Ghrh*^+^ cells after vehicle (0x) or single (1x) or repeated (5x) i.p. 2DG administration or repeated 2DG administration with mdivi-1 treatment (5x MD). *P = 0.04 0x vs. 1x; ^#^*P* = 0.01 0x vs. 5x MD, Welch’s *F*_[3, 7.869]_ = 8.338, Welch’s ANOVA test with Dunnett’s multiple-comparisons test, *n* = 4–9/group. Each dot represents data from individual cells or animals and data are displayed as mean ± SEM.

To address whether maintaining mitochondrial structure could preserve sensitivity to low glucose in cells repeatedly treated with low glucose, we used the DRP1 inhibitor mdivi-1. Mdivi-1 inhibits mitochondrial fission in many mammalian cells (37). Mdivi-1 maintained mitochondrial length (Figure 10A) and preserved low glucose–induced depolarization in N38 cells treated with repeated glucose deprivation (Figure 10B). In vivo, combined daily treatment with 2DG and mdivi-1 partially reversed the blunted glucose response to repeated glucose deprivation (Figure 10C) and restored GHRH neuron activation (Figure 10D). These findings suggest that mdivi-1 treatment blunts mitochondrial fission and inhibiting mitochondrial fission in vivo maintains GHRH neuron sensitivity to glucose deprivation even after repeated episodes.

## Discussion

The CRR to hypoglycemia is multifaceted, with behavioral, neural, and hormonal components. Our studies indicate GHRH neurons may contribute to several aspects of the CRR. Hypoglycemia increases food intake and recent reports show that non-AGRP, non-POMC ARC neurons play a role in feeding (38, 39). Our data demonstrate that GHRH neurons are GABAergic, non-AGRP, non-POMC ARC neurons and activated by hypoglycemia and so could contribute to feeding response. The effects of repeated 2DG or insulin hypoglycemia on feeding in mice are unknown, but in rats, repeated 2DG blunts the feeding response (23), whereas repeated insulin-induced hypoglycemia has no effect (40). GHRH neuron activity is decreased by both repeated 2DG and insulin-induced hypoglycemia, suggesting cells other than GHRH neurons likely maintain feeding with repeated insulin hypoglycemia (10, 41). We also found that GHRH neurons are synaptically connected to the pancreas, so hypoglycemia-activated GHRH neurons could directly influence pancreatic endocrine function. Although our tracing studies show few GHRH neurons are labeled by retrograde tracing from the pancreas, this is likely to be an underestimate because we are only able to target PRV injections to a fraction of the pancreas. Further work examining the feeding and hormonal responses to GHRH neuron modulation is needed to determine their roles in hypoglycemia-induced feeding and pancreatic hormone release.

Both insulin hypoglycemia and 2DG increased GHRH neuron activity, which was blunted by repeated treatment. In addition, both repeated 2DG and insulin hypoglycemia significantly decreased (*P* < 0.05) GHRH spine number, suggesting diminished excitatory input. Together, these results suggest GHRH neuron activation with acute glucose deprivation, reduced GHRH activation, and fewer excitatory inputs into GHRH neurons with repeated glucose deprivation are consequences of glucose metabolism. GHRH activation was greater with 2DG than with insulin hypoglycemia. 2DG treatment resulted in hyperglycemia, and osmotic changes with hyperglycemia contribute to glucose sensing in β cells (42), so hyperglycemia and osmotic changes may lead to greater GHRH neuron activation with 2DG treatment.

2DG and insulin treatment have differing effects on SST^+^ inputs to GHRH neurons and on plasma GH. Repeated 2DG increased SST^+^ terminals on GHRH neurons, but SST^+^ terminals on GHRH neurons were unchanged with repeated insulin hypoglycemia. Insulin-induced hypoglycemia increased plasma GH in mice in keeping with the findings in humans, where insulin-induced hypoglycemia is the gold standard test for somatotrophic axis function in clinical practice (43). However, acute 2DG treatment did not significantly alter plasma GH. Together, these findings suggest insulin or peripheral hyperglycemia may have effects on SST neurons and pituitary somatotrophs to control GH release in addition to the effects of low glucose on GHRH neurons. For example, SST is elevated by low glucose and 2DG administration in rats (44) and can suppress pituitary GH release as well as override GHRH. Insulin receptors are expressed on SST neurons (45), and insulin-induced hypoglycemia in combination with GHRH treatment has a greater effect on plasma GH than either alone. In clinical studies, insulin-induced hypoglycemia increases GH even in individuals with GHRH insensitivity (46). These data suggest insulin may increase GH independent of GHRH release. Hyperglycemia can also suppress pituitary GH release (47). Other neurotransmitters and hormones may also contribute to the differences in GH response to insulin and 2DG. GABA, which colocalizes with GHRH in our studies, inhibits GH release (48). Both insulin and glucose regulate plasma ghrelin, which can directly and indirectly regulate pituitary GH secretion (49, 50).

The importance of GH release in the CRR to hypoglycemia is unclear and species differences exist. In rats, hypoglycemia reduces plasma GH (51). In sheep, GH increases with hypoglycemia in the fasted but not fed state (52). In mice, hypoglycemia has been reported to decrease (53) or have no effect (54) on GH. In humans, increased GH occurred in a small proportion of patients with spontaneous hypoglycemia (<2.2 mmol/L or 40 mg/dL) independent of insulin levels (55). However, hypoglycemia is a common complication of GH deficiency (56). There is some evidence that GHRH neurons may contribute to the CRR beyond GH release. Magel2-null mice have defects in the hypothalamic regulation of GH (as shown by a blunted GH response to ghrelin) but normal pituitary function (i.e., GH response to exogenous GHRH is normal). These mice have more profound hypoglycemia with insulin treatment than control animals (57). In our studies, GHRH neuron activation is blunted by repeated 2DG or insulin treatment, suggesting that decreased GHRH activity may contribute to impaired CRR with repeated hypoglycemia (58). Dissecting the contribution of GHRH neurons to the CRR independent of GH will require examination of the CRR response in GHRH-deficient mice, with and without GH replacement. Longitudinal analyses and examination of the direct effects of 2DG and insulin on pituitary GH release will provide additional insights into this aspect of counterregulation in mice.

Repeated glucose deprivation shifted the balance of excitatory and inhibitory inputs on GHRH neurons. Excitatory dendritic spines were absent, inhibitory SST terminals were markedly increased, and these changes were associated with microglial activation, known to be involved in synaptic remodeling (59). Electrophysiological assessment of excitatory and inhibitory postsynaptic currents in response to acute and repeated glucose deprivation would confirm these changes in GHRH neuron inputs. Similar alterations in inputs occur in ARC AGRP neurons with nutritional status (60), and repeated hypoglycemia leads to synaptic remodeling in VMH neurons (61). Although GHRH neurons are known to have both glutamatergic and GABAergic inputs, the sites of these neural inputs are unknown. Other ARC neural populations have inputs from multiple hypothalamic, midbrain, and other brainstem regions (62), and the multipolar morphology of GHRH neurons is typical of integrative neurons. Mapping inputs onto GHRH neurons would be the next step in determining how GHRH neurons integrate glucose sensing.

In keeping with our previous studies (10), GHRH neurons were activated by low glucose in vivo. This is likely via cell-autonomous glucose sensing because there were no significant changes in dendritic spines or SST terminals with acute glucose deprivation. We cannot exclude alterations in the strength of excitatory inputs that occurred without dendritic spine remodeling or in non–SST-expressing inhibitory inputs. We developed an in vitro model of hypoglycemia insensitivity to dissect the cell-autonomous changes that occur with repeated hypoglycemia. N38 cells have an embryonic origin and express *Agrp*, *Pomc*, and *Ghrh*. The embryonic origin of the cell line means these cells may not completely replicate GHRH neurons in adult mice. However, they recapitulated many of the in vivo responses in GHRH neurons, including activation and increased GHRH with low glucose, and a blunted calcium and GHRH response with repeated low glucose. Our findings are similar to those observed in ventromedial hypothalamus (VMH) GI neurons, with low glucose–induced activation that is blunted by previous hypoglycemia (20). Calcium influx in hypothalamic GI neurons is via voltage-gated calcium channels, and blunted calcium influx could occur through hyperpolarization or depolarization block (63). Our voltage imaging studies suggest that repeated low glucose leads to hyperpolarization of N38 cells, rather than depolarization block. Previous work has implicated ATP-sensitive chloride channels such as the cystic fibrosis transmembrane receptor (CFTR) in the silencing of GI neurons with increased glucose (64, 65). Increased intracellular ATP, which we observed with repeated low glucose, would open CFTR to increase chloride currents, leading to hyperpolarization even when glucose is low.

Mitochondrial dynamics are critical to glucose sensing in hypothalamic GE neurons (66). In VMH GE neurons, high glucose reduces ROS and mitochondrial length, leading to systemic glucose abnormalities (67). Similarly in ARC GE neurons, genetic disruption of mitochondrial remodeling alters ARC glucose sensing, resulting in disrupted glucose metabolism (13). We have extended these findings by demonstrating that mitochondrial dynamics contribute to glucose sensing in GI neurons. Similar to previous studies that show a role for disrupted mitochondrial dynamics in abnormal glucose metabolism secondary to high-fat diet, our data support the concept that mitochondrial abnormalities contribute to the blunted glucose response with repeated glucose deprivation. Therefore, abnormal mitochondrial structure may be a common mechanism leading to reduced glucose sensing with prolonged hyper- or hypoglycemia. Additional factors likely regulate mitochondrial dynamics in response to low glucose. For example, uncoupling protein 2 (UCP2) regulates mitochondrial fission and fusion in ARC and VMH neurons with high glucose (67, 68). Further studies are needed to assess the role of UCP2 or other pathways in mitochondrial remodeling with hypoglycemia in GHRH and other GI neurons.

Targeting mitochondrial fission may provide a strategy to prevent HAAF. In VMH and ARC GE neurons, genetic restoration of mitochondrial remodeling restores glucose sensing and normalizes blood glucose (13, 67). Here we chose to reduce mitochondrial fission with a pharmacological approach. Our data suggest repeated hypoglycemia disrupts multiple points in the glucose-sensing network: glucose sensing in GHRH neurons as well as their inputs. Systemic pharmacological treatment would act at multiple sites and might have translational applications beyond genetic manipulations. The DRP1 inhibitor mdivi-1 preserved mitochondrial length and maintained responsiveness to low glucose after repeated episodes in vitro and in vivo. Our in vitro findings suggest mdivi-1 improves glucose sensing through intracellular effects, but it may also restore the balance of excitatory and/or inhibitory inputs into GHRH neurons. Systemic mdivi-1 has been reported to improve synaptic function in neurodegenerative diseases (69); however, detailed assessments are needed to determine its exact mechanism. Mdivi-1 inhibits mitochondrial fission via actions on DRP1, but DRP1 plays roles in multiple downstream pathways (70). Mdivi-1 has also been reported to have DRP1-independent effects on mitochondrial complex I to attenuate ROS production (71). Further studies are required to understand and selectively target the DRP-1–dependent and –independent pathways involved and determine their contributions to preventing HAAF.

In conclusion, GHRH neurons form a distinct population of GABAergic ARC neurons that are synaptically connected to the pancreas. These neurons are activated by low glucose in vivo. Repeated glucose deprivation blunts GHRH neuron activation through circuit-wide and cell-intrinsic effects. Excitatory inputs are diminished and inhibitory inputs upregulated with marked microglial activation in vivo. Using a new in vitro model of blunted glucose sensing, we show that repeated glucose deprivation reduces GHRH release, diminishes calcium influx, and hyperpolarizes GHRH-expressing N38 cells. Repeated glucose deprivation blunts the effects of low glucose on ATP production and mitochondrial elongation. Maintaining mitochondrial length by pharmacological inhibition of mitochondrial fission preserves responsiveness to hypoglycemia in vitro and in vivo even after repeated episodes. These studies demonstrate attenuation of glucose sensing and modification of GHRH inputs with repeated hypoglycemia to form a foundation for future studies that specifically target GHRH activity and mitochondrial function in GHRH neurons. Together, our findings reveal that structural and functional remodeling of hypothalamic circuits and glucose-sensing neurons contribute to decreased responsiveness to low glucose and suggest that targeting mitochondrial dynamics may offer new approaches to prevent HAAF.

## Methods

### Animals.

Male and female C57BL/6J and GHRH-GFP heterozygous mice (C57BL/6J background) (12–30 weeks) were housed under controlled conditions (12-hour light/12-hour dark cycle, 22°C) and fed ad libitum on standard mouse chow. GHRH-GFP mice were a gift from Iain Robinson (Medical Research Council National Institute for Medical Research, London, United Kingdom).

### In vivo studies.

Animals were randomized by body weight. Animals were fasted (5 hours) in their home cage before treatment with i.p. saline, 2DG (MilliporeSigma, 400 mg/kg), or human insulin (Humulin R, Lilly, 0.5 mU/kg). Animals were treated on 5 consecutive days receiving saline alone (0x), saline for 4 days and 2DG/insulin on day 5 (acute deprivation, 1 episode) or 2DG/insulin for 5 days (repeated glucose deprivation, 5 episodes). To assess the effects of inhibition of mitochondrial fission in vivo, mice were treated simultaneously with 2DG and mdivi-1 (MilliporeSigma, 40 mg/kg in corn oil) or vehicle. Glucose readings were taken from tail blood at 90 minutes. For ISH studies, mice were sacrificed 30 minutes after final injection. For measurement of CRR hormones, retro-orbital blood was taken 90 minutes after injection. Blood samples were centrifuged (15,000 rpm, 4°C, 10 minutes), and plasma was stored at –80°C until analysis.

For retrograde tracing studies, GHRH-GFP mice were anesthetized with isoflurane before injection of PRV-RFP (5 × 100 nL) into the epigonadal fat pad, pancreas, gastrocnemius muscle, adrenals, or liver. After injection, the needle was left in place for 10 minutes. The skin was closed with stainless steel clips and appropriate analgesia given. Mice were euthanized 5–7 days later.

### Mouse brain processing.

Unless noted otherwise, isoflurane-anesthetized mice were transcardially perfused with 0.9% saline followed by 4% paraformaldehyde (PFA) in 0.1 M phosphate buffer (Electron Microscopy Sciences). Brains were removed and immersed in 4% PFA at 4°C overnight.

### Assays.

Blood glucose was determined using a Breeze 2 glucometer (Bayer). Plasma levels of CRR hormones and GHRH levels in cell supernatant were determined by ELISA (Crystal Chem Glucagon 81518; Corticosterone 80556; GH Millipore Sigma 3P EZRMGH45K; GHRH Aviva Systems Biology OKEH03121).

### Iontophoretic dye injection, neuronal imaging, 3D neuron reconstruction, and morphological analysis.

Ninety minutes after the final treatment, GHRH-GFP mice were perfused with PBS, then 1% PFA, followed by 4% PFA/0.125% glutaraldehyde in phosphate buffer of neutral pH. Brains were removed and postfixed in the same fixative for 6 hours. Coronal sections (125 μm) were cut using a vibratome (Leica VT1000S) and mounted on a nitrocellulose membrane filter in phosphate buffer. Using an epifluorescence microscope (Leica DM LFS) equipped with a micromanipulator and a current generator, GHRH-GFP–expressing neurons were identified for iontophoretic dye injection under a ×40 objective. Once identified, a glass pipette filled with 5% Alexa Fluor 555 dye was used to penetrate the neuronal cell body, and a direct current (5–10 nA) was applied to ensure filling of the entire cell. Per section, 1~3 cells were injected per section, spaced so as to prevent overlap of their dendrites. Tissue sections with filled cells were mounted onto SuperFrost slides using Fluoromount G and coverslipped using spacers. Images of dendritic spines were captured and analyzed as previously described (72), from dendritic segments selected at 0–200 μm from the soma. The selection criteria for dendritic spines were as previously described (73). The investigator was blinded to treatment groups. Dendritic segments from 29 neurons were examined, with an average of 2 neurons from 16 animals, yielding 656 μm of dendritic processes quantified. Confocal images were deconvolved using an iterative blind deconvolution algorithm (AutoQuant X3) and analyzed manually for spine density and classification after volumetric reconstruction using Imaris software (Bitplane). Spines were classified into filopodia/thin, stubby, and mushroom based on the head-to-neck diameter ratio and maximum head diameter.

### iDISCO+ brain clearing.

iDISCO+ was performed as described previously (21). In brief, brains were dehydrated with a methanol gradient (20%–100%) at room temperature (RT), incubated in 100% dichloromethane (DCM) (MilliporeSigma), and bleached in 5% H_2_O_2_ overnight at 4°C. Brains were then rehydrated with a methanol gradient (80%–20%) and incubated in permeabilization buffer with 5% DMSO, 0.3 M glycine in 0.1% Triton X-100, 0.05% Tween-20, 0.0002% heparin, and 0.02% NaN_3_ (PTxwH) in 1× PBS for 1 day. Brains were incubated in blocking buffer (PTxwH with 3% normal donkey serum from Jackson ImmunoResearch Laboratories Inc) at 37°C overnight. Immunolabeling for GFP (Aves, Table 1) was performed in blocking buffer (4 days, 37°C) and was followed by 5 washes in PTxwH and incubation with goat anti-chicken antibody (Life Technologies, Thermo Fisher Scientific, A11039) (4 days, 37°C). Samples were washed in PTxwH at 37°C and in PBS at RT. Optical clearing was achieved by dehydrating with a methanol gradient (20%–100%), washing in 100% methanol followed by DCM, before transferring the samples to dibenzyl ether (MilliporeSigma). The hypothalamus was imaged with an Ultramicroscope II (LaVision BioTec) using a ×4 objective, with *Z*-stacks acquired at a *Z*-step of 3 μm, giving an average of 492 sections per animal. Hypothalamic images were analyzed with Imaris for quantification of GFP-positive neurons using the Imaris “spot” detection algorithm. The count was not corrected to the brain volume.

### Immunocytochemistry, IHC, and ISH.

For immunocytochemistry (ICC), cells grown on fibronectin-coated coverslips were washed twice in PBS and then fixed for 15 minutes in 4% PFA (Electron Microscopy Services). For IHC, coronal slices were cut at 50 μm thickness on a vibratome (Leica VT1000). Immunofluorescence and double labeling were performed as previously described (10). Briefly, cells or tissue were washed in 0.03 M Triton X-100 (TX) in PBS followed by incubation in blocking solution (1 hour, 3% bovine serum albumin in 0.03 M TX/0.1 M PBS). Cells or tissue sections were incubated in primary antibody in blocking solution (16–72 hours, 4°C; see Table 1). Cells or tissue sections were rinsed in TX/PBS and incubated in blocking solution containing secondary antibody (Alexa Fluor conjugated, Life Technologies, Thermo Fisher Scientific, 1:1000, 2 hours, RT, catalog numbers A11032, A11034, and A11039). After staining, cells or tissue sections were mounted using Fluoromount (Southern Biotech). For IHC to detect NPY, POMC, and AGRP, GHRH-GFP mice were treated with colchicine to enhance cell body staining, as previously described (10).

For ISH, brains were snap-frozen in liquid nitrogen, and 20 μm sections were cut on a cryostat. An RNAscope multiplex fluorescence assay kit was used (Advanced Cell Diagnostics; catalog 323100). Briefly, sections were fixed in 4% PFA for 1 hour at 4°C, then dehydrated in ethanol followed by protease III treatment for 30 minutes. Sections were hybridized with RNAscope oligonucleotide probes against *Ghrh* (470991), *Gck* (400971), *Sst* (404631), *fos* (316921), *Gad2* (439371), and/or *Slc17a6* (319171), then incubated with signal amplifier and TSA Plus fluorophores (PerkinElmer Inc.; catalog NEL744001KT, NEL745001KT, NEL741001KT) according to RNAscope protocols. Slides were washed with RNAscope wash buffer, counterstained with DAPI, and mounted with ProLong Gold mounting medium (Thermo Fisher Scientific).

Images were acquired as *Z*-stacks using a Zeiss Inverted LSM 780 laser scanning confocal microscope (Carl Zeiss MicroImaging, Inc.). For quantification purposes, exposure times were set based on the control group and were comparable among the different experimental groups. Staining intensity, number of cells, and colocalization were quantified using the Imaris software and profile-based counting method. The investigator was blinded to treatment groups. Every third section for IHC and every sixth section for ISH was collected, stained, and imaged.

Confocal image stacks were reconstructed and visualized as 3D volumes with Imaris software. The Imaris “spot” detection algorithm was used for semiautomatic identification, counting of fluorescently labeled cells, and colocalization of cells and of terminals close to GHRH neurons. We considered spots that were within 0.5 μm to be colocalized. To quantify gene expression signal, the area and intensity of more than 20 dots were measured to determine the mean intensity per dot. Regions of interest (ROIs) were drawn around each *Ghrh* neuron, and the total signal area and intensity in each ROI measured were then normalized for the mean intensity per dot.

### Quantitative real-time PCR.

Total RNA was extracted from N38 cells using RNeasy Plus Kit (QIAGEN) according to the manufacturer’s protocol. RNA purity and quantity were assessed by spectrophotometry (Spectramax, Molecular Devices), and real-time PCR was performed using an ABI 7500 system as previously described (10) using the following primers: GHRH: primer 1 CTGTATGCCCGGAAAGTGAT, primer 2 GTCATCTGCTTGTCCTCTGTC, and probe TCCTCTCCC. RPL23: primer 1 TTAGCTCCTGTGTTGTCTGC, primer 2 ACTTCCTTTCTGACCCTTTCC, and probe TTCGACATCTTGAACG.

### Cell culture and in vitro studies.

Murine hypothalamic neural cells (N38, Cellutions Biosystems, *Mycoplasma* testing and short tandem repeat profiling performed by Cellutions Biosystems) were grown in normal culture medium comprising high glucose (25 mM) DMEM/10% FBS at 37°C and 5% CO_2_. For ICC studies, cells were grown on fibronectin-coated 12 mm cover glass (Thermo Fisher Scientific).

For acute or repeated glucose deprivation, culture medium was replaced with no-glucose DMEM/10% FBS supplemented with 25 mM (control) or 2.5 mM glucose (glucose deprivation) for 2 hours. To assess the effects of glucose deprivation at lower glucose concentrations, culture medium was replaced with no-glucose DMEM/10% FBS supplemented with 5 mM (control) or 0.5 mM glucose (glucose deprivation) for 2 hours. Normal culture medium was replaced at the end of this period. Cells were treated with no glucose deprivation (5 treatments with 25 mM glucose/DMEM/10% FBS), acute glucose deprivation (1 episode, 4 treatments with 25 mM glucose/DMEM/10% FBS and 1 treatment with 2.5 mM glucose/DMEM/10% FBS), repeated glucose deprivation (3 episodes: 2 treatments with 25 mM glucose/DMEM/10% FBS and 3 treatments with 2.5 mM glucose/DMEM/10% FBS; or 5 episodes: 5 treatments with 2.5 mM glucose/DMEM/10% FBS). A further set of cells were treated using 5 mM and 0.5 mM glucose in place of 25 mM and 2.5 mM glucose. To assess the effects of inhibiting mitochondrial fission, N38 cells were treated as described above with the addition of mdivi-1 (50 nM) or DMSO (control). At the end of the final treatment period, either supernatant was removed and stored at –80°C for GHRH assay, with cells fixed for ICC, or cells were lysed in RNA lysis buffer (QIAGEN) and the lysate stored at –80°C until RNA purification. Each study was repeated on at least 3 occasions with 4 replicates. For examination of mitochondrial membrane potential, N38 cells were incubated with MitoTracker Red CMXRos (100 nM, Invitrogen, Thermo Fisher Scientific) in culture medium for the final 30 minutes of the last treatment period. Cells were fixed in 4% PFA in culture medium for 15 minutes before rinsing in PBS and mounting (Fluoromount with DAPI). Confocal images were acquired as described above. Fluorescence intensity was quantified using Fiji software with ROI over individual cells.

### Calcium and voltage imaging.

For calcium imaging studies, N38 cells grown on fibronectin-coated cover glass were loaded with Fluo-4 (Invitrogen, Thermo Fisher Scientific) in Tyrode’s solution (140 mM NaCl, 5 mM KCl, 5 mM HEPES, 1 mM NaH_2_PO_4_, 1 mM MgCl_2_, 1.8 mM CaCl_2_, 25 mM glucose) as previously described (74). Cells were imaged in calcium imaging buffer (74) with 25 mM, 5 mM, or 2.5 mM glucose using a Zeiss Axio Observer Z1 inverted widefield fluorescence microscope. Images were acquired every 6 seconds for 3 minutes at a resolution of 1024 × 1024 pixels with a ×10 Plan-Apochromat objective. For voltage imaging studies, N38 cells grown on chamber slides (Ibidi) were washed twice, then loaded with Fluovolt (Invitrogen, Thermo Fisher Scientific) in Tyrode’s solution according to the manufacturer’s instructions. Imaging was performed in Tyrode’s solution with 25 mM, 5 mM, or 2.5 mM glucose in 5% CO_2_ at 37°C. Images were acquired every second for 180 seconds at a resolution of 512 × 512 pixels using a Zeiss LSM780 confocal microscope with a ×20/1.4 NA Plan-Apochromat objective. All imaging studies were performed on 3 occasions for each condition. Fluorescence intensity was quantified using Fiji software with ROIs over individual cells (calcium imaging) or cell membranes (voltage imaging).

### Measurement of OCR.

OCR, ECAR, ATP production, maximal respiration, and spare respiratory capacity were determined in a Seahorse XFe24 extracellular flux analyzer. Measurements were performed at the end of the final glucose deprivation treatment in medium with 10 mM glucose and 1% FBS. In preliminary studies, OCR and ECAR measurements in 25 mM glucose were at the lower limit of detection. Basal measurements were determined, and OCRs were recorded after the following additions: oligomycin (2.5 μmol/L), carbonyl cyanide 4-(trifluoromethoxy)-phenylhydrazone (1 μmol/L), rotenone (1 μmol/L), and antimycin A (1 μmol/L).

### Measurement of ROS.

For measurement of ROS, N38 cells were cultured on 96-well plates and treated as described above. For the final hour of treatment, Amplite ROS Green (AAT Bioquest) was added to the culture medium. Fluorescence signal was measured by microplate reader (Spectramax, Molecular Devices).

### Statistics.

Data are shown as mean ± SEM. Distribution was assessed by the Kolmogorov-Smirnov test and Shapiro-Wilk test. Significance was determined by the unpaired 2-tailed *t* test, 1-way ANOVA with the post hoc Tukey’s (Gaussian distribution), Welch’s ANOVA (Gaussian distribution with unequal standard deviation), Kruskal-Wallis test (nonparametric distribution), or 2-way ANOVA with Holm-Šidák multiple-comparisons test for repeated measures. Significance was set at α = 0.05.

### Study approval.

Animal care and experimental procedures were performed with the approval of the Institutional Animal Care and Use Committee of Icahn School of Medicine at Mount Sinai under established guidelines.

## Author contributions

MB performed experiments, analyzed data, and contributed to the writing of the manuscript. AA, KD, RL, MJG, DG, and KC performed experiments and reviewed the manuscript. MV, MNS, JEC, and PRH provided experimental and intellectual expertise. SAS performed experiments, analyzed data, and wrote the manuscript. MB, AA, KD, and SAS designed the studies. All authors discussed the results and edited the manuscript.

## Supplementary Material

supplemental data

## Figures and Tables

**Table 1:**
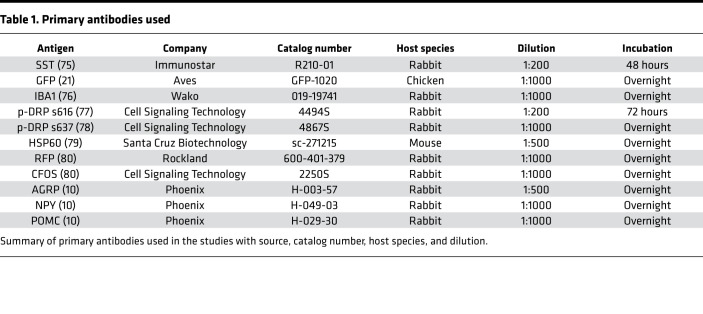
Primary antibodies used

## Version 1 11/05/2020

Electronic publication
