# Supplementary material for: Repeated hypoglycemia remodels neural inputs and disrupts mitochondrial function to blunt glucose-inhibited GHRH neuron responsiveness
Source: JCI Insight. 2020 Nov 5;5(21):e133488. doi: 10.1172/jci.insight.133488 (PMC7710320; doi:10.1172/jci.insight.133488)
Supplement: supplemental data [file jciinsight-5-133488-s098.pdf]

Figure S1

A

	DAYS 1-4	DAY 5
0X	Saline	Saline
1X	Saline	2-Deoxy-D -Glucose
5X	2-Deoxy-D -Glucose	2-Deoxy-D -Glucose

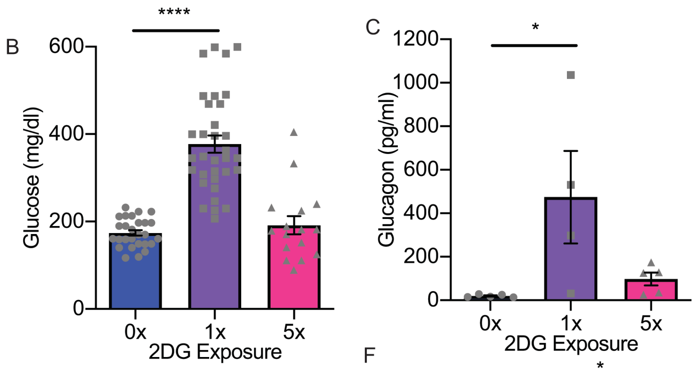

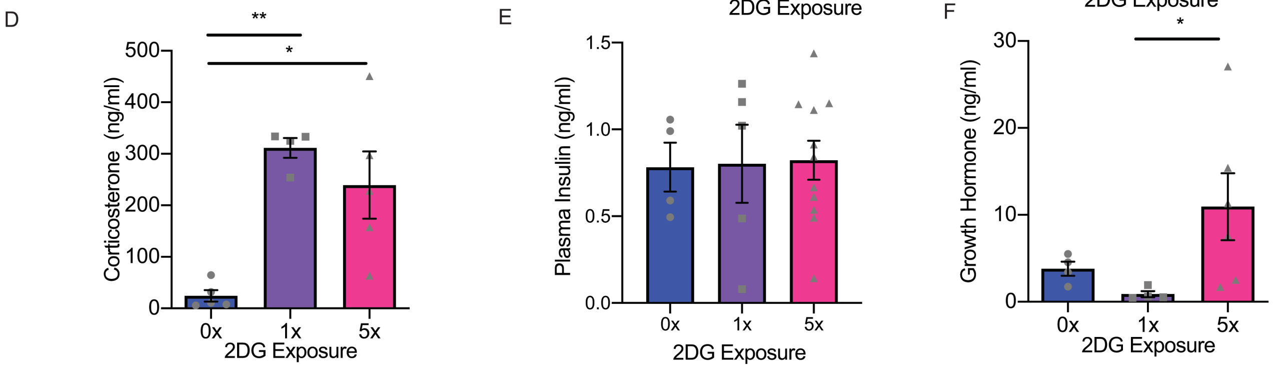

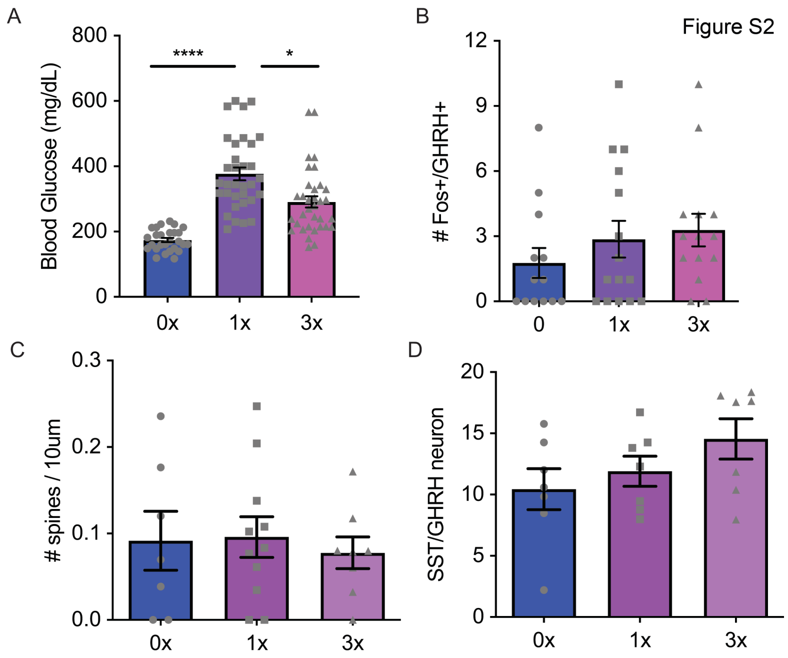
Figure S2
A
Blood Glucose (mg/dL)
800
600
400
200
0
****
*
0x
1x
3x
B
Fos+/GHRH+
12
9
6
3
0
0
1x
3x
C
spines / 10um
0.3
0.2
0.1
0.0
0x
1x
3x
D
SST/GHRH neuron
20
15
10
5
0
0x
1x
3x

Figure S3

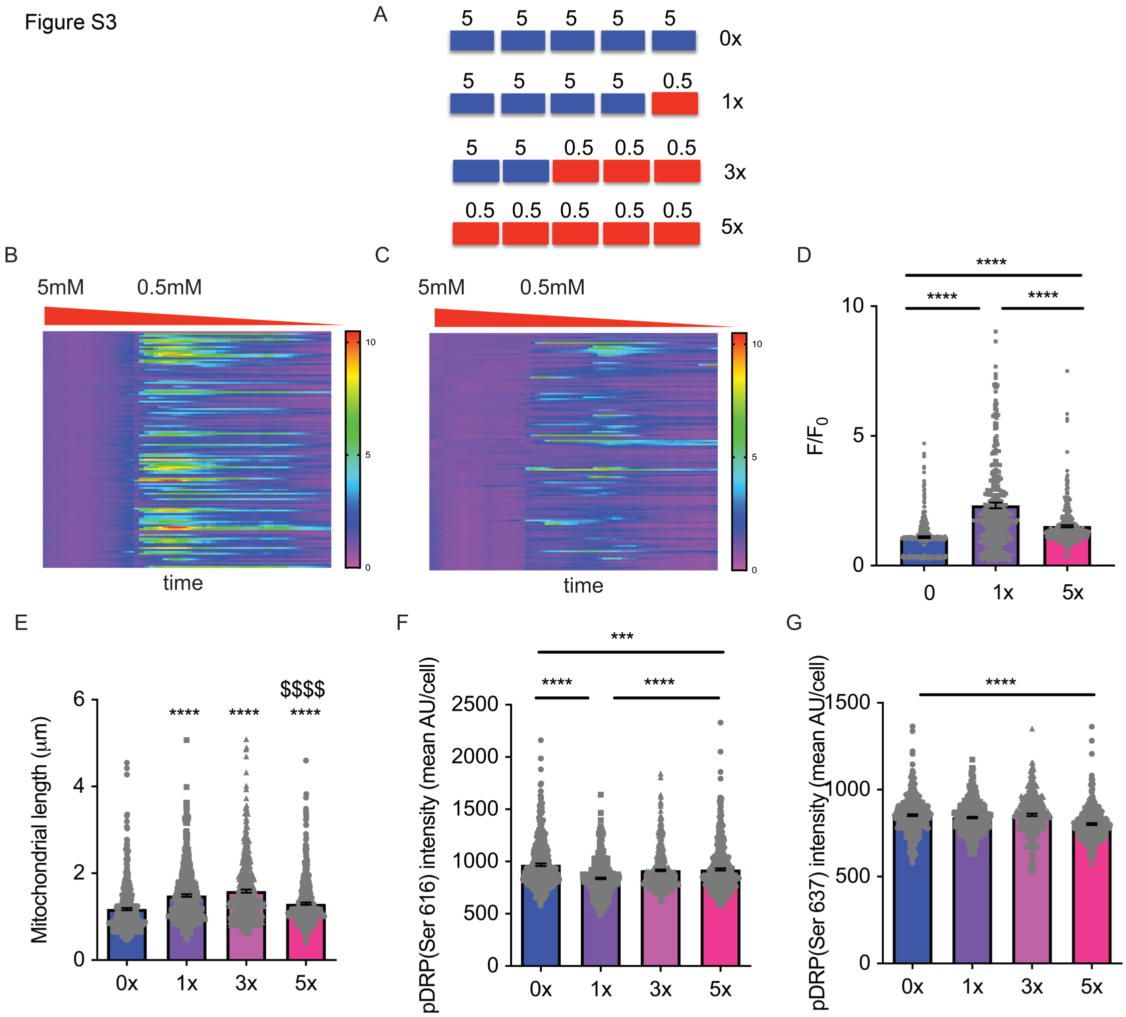

A
5 5 5 5 5 0x
5 5 5 5 0.5 1x
5 5 0.5 0.5 0.5 3x
0.5 0.5 0.5 0.5 0.5 5x
B
5mM 0.5mM
time
C
5mM 0.5mM
time
D
F/F0
0 1x 5x
E
Mitochondrial length (μm)
$$$$
0x 1x 3x 5x
F
pDRP(Ser 616) intensity (mean AU/cell)
0x 1x 3x 5x
G
pDRP(Ser 637) intensity (mean AU/cell)
0x 1x 3x 5x
